# Predicting survival in patients with SARS-CoV-2 based on cytokines and soluble immune checkpoint regulators

**DOI:** 10.3389/fcimb.2024.1397297

**Published:** 2024-11-25

**Authors:** Nuri Lee, Kibum Jeon, Min-Jeong Park, Wonkeun Song, Seri Jeong

**Affiliations:** ^1^ Department of Laboratory Medicine, Hallym University College of Medicine, Kangnam Sacred Heart Hospital, Seoul, Republic of Korea; ^2^ Department of Laboratory Medicine, Hallym University College of Medicine, Hangang Sacred Heart Hospital, Seoul, Republic of Korea

**Keywords:** immune checkpoint, cytokines, SARS-CoV-2, machine learning, prognosis

## Abstract

**Background:**

Coronavirus disease 2019 (COVID-19) has been widespread for over four years and has progressed to an endemic stage. Accordingly, the evaluation of host immunity in infected patients and the development of markers for prognostic prediction in the early stages have been emphasized. Soluble immune checkpoints (sICs), which regulate T cell activity, have been reported as promising biomarkers of viral infections.

**Methods:**

In this study, quantitative values of 17 sICs and 16 cytokines (CKs) were measured using the Luminex multiplex assay. A total of 148 serum samples from 100 patients with COVID-19 were collected and the levels were compared between survivors vs. non-survivors and pneumonic vs. non-pneumonic conditions groups. The impact of these markers on overall survival were analyzed using a machine learning algorithm.

**Results:**

sICs, including sCD27, sCD40, herpes virus entry mediator (sHVEM), T-cell immunoglobulin and mucin-domain containing-3 (sTIM-3), and Toll-like receptor 2 (sTLR-2) and CKs, including chemokine CC motif ligand 2 (CCL2), interleukin-6 (IL-6), IL-8, IL-10, IL-13, granulocyte-macrophage colony-stimulating factor (GM-CSF), and tumor necrosis factor-α (TNF- α), were statistically significantly increased in the non-survivors compared to those of in the survivors. IL-6 showed the highest area under the receiver-operating curve (0.844, 95% CI = 0.751–0.913) to discriminate non-survival, with a sensitivity of 78.9% and specificity of 82.4%. In Kaplan-Meier analysis, patients with procalcitonin over 0.25 ng/mL, C-reactive protein (CRP) over 41.0 mg/dL, neutrophil-to-lymphocyte ratio over 18.97, sCD27 over 3828.8 pg/mL, sCD40 over 1283.6 pg/mL, and IL-6 over 21.6 pg/mL showed poor survival (log-rank test). In the decision tree analysis, IL-6, sTIM-3, and sCD40 levels had a strong impact on survival. Moreover, IL-6, CD40, and CRP levels were important to predict the probability of 90-d mortality using the SHapley Additive exPlanations method.

**Conclusion:**

sICs and CKs, especially IL-6, sCD27, sCD40, and sTIM-3 are expected to be useful in predicting patient outcomes when used in combination with existing markers.

## 1 Introduction

As the SARS-CoV-2 pandemic has been prevalent for more than four years, it is progressing to the endemic stage, where SARS-CoV-2 variants occur and the number of re-infected patients increases (Markov et al., 2023; Nesteruk, 2023; Nguyen et al., 2023). To respond to this ongoing endemic stage of coronavirus disease 2019 (COVID-19), control of the infection from a long-term perspective has been emphasized and the importance of host immunity in each patient has increased. In addition, laboratory tests are important not only for the detection of SARS-CoV-2 infection but also for the screening and prognosis prediction of severe patients (Carabelli et al., 2023; Lim, 2023; Puhach et al., 2023). Many studies have been conducted on infection markers in SARS-CoV-2 patients (Kermali et al., 2020). C-reactive protein (CRP), white cell count, and cytokines (CK) such as interleukin-6 (IL-6), IL-8, and IL-10 have been investigated as factors associated with the diagnosis or severity of COVID-19 (Han et al., 2020; Kermali et al., 2020). CRP is a plasma protein generated in the liver in response to inflammatory mediators and is associated with disease severity during SARS-CoV-2 infection. Regarding white blood cells (WBC), in patients with severe COVID-19, there is a pattern where the neutrophil count is dominant and the lymphocyte count decreases, leading to the expectation that the neutrophil-to-lymphocyte (N-to-L) ratio could serve as a potential biomarker for the early detection of severe features. In particular, IL-6, which functions as a pleiotropic CK with both pro- and anti-inflammatory functions, displays hyperinflammation beyond homeostatic levels, inducing CK release syndromes, such as a CK storm (REMAP-CAP Investigators, 2021). The elevation of IL-6 in both serum and alveoli serves as an indicator of hyperinflammation and is utilized as an unfavorable prognostic factor (REMAP-CAP Investigators, 2021). In addition, soluble immune checkpoints (sICs) are emerging factors for understanding SARS-CoV-2 infection and progression (Cai et al., 2020; Sun et al., 2022). Previous studies have reported the importance of sICs as potential biomarkers of viral infections (Cai et al., 2020; Aghbash et al., 2021; Morrell et al., 2022). sICs are expressed on immune cells to regulate T cell activity and primarily act as immune evasion mechanisms (Marin-Acevedo et al., 2018; Zahavi and Weiner, 2019; Cai et al., 2020; Joseph et al., 2021; Sun et al., 2022; Yu et al., 2023). Specifically, in SARS-CoV-2 infection, up-regulation of T-cells has been reported in several studies; sICs are implicated in mediating T-cell exhaustion and inducing T-cell lymphopenia, critical factors contributing to the pathogenesis of severe COVID-19 (Cai et al., 2020). However, few studies have quantitatively evaluated the effectiveness of sICs as prognostic markers in clinical practice for patients with SARS-CoV-2 infection (Gambichler et al., 2020; Gatto et al., 2020; Awadasseid et al., 2021). Additional research is required to concurrently assess various biomarkers, determine markers related to host immunity, and predict prognosis in the early stages of SARS-CoV-2 infection.

In a previous study, the authors compared the quantitative values of sICs and CKs in SARS-CoV-2 infected patients in the survivor and non-survivor groups (Lee et al., 2022). However, this study has some limitations. The number of target patients was low, and some specimens fell below the limit of detection (LoD), making it challenging to measure CK levels accurately. In the current study, we aimed to enhance the robustness of our findings by implementing several methodological enhancements. Firstly, we doubled the total number of patients with SARS-CoV-2 and focused on samples collected within one week of SARS-CoV-2 diagnosis. The focus on the first week was paramount as it represented a critical period for patient management. Furthermore, we took measures to ensure adequate sample volume from all patients, thereby reducing instances of the samples falling below the LoD. Additionally, our study enrolled patients during the period when the Omicron variant was prevalent. The variant exhibited distinct clinical characteristics, including different mortality rates, compared to the Wuhan strain variant in the previous study. Secondly, infection markers, including WBC, N-to-L ratios, and CRP (Ponti et al., 2020; Bedel and Korkut, 2021; Agarwal, 2022), were added and analyzed in an integrated manner along with sICs and CK. Furthermore, co-infection with fungi, bacteria, and viruses in patients with SARS-CoV-2 infection has a poor prognosis, and the microbial community interacts with immune responses related to prognosis (Jeong et al., 2022). Jeong et al. reported that SARS-CoV-2 infection is related to multidrug resistance (Jeong et al., 2022). Therefore, this study sought to examine the relationship between sICs and multidrug-resistant bacteria. Finally, we utilized a machine learning (ML) algorithm to efficiently evaluate the importance of various types of biomarkers in prognosis prediction.

In particular, this study emphasizes the utilization of sICs and CKs in clinical practice and seeks to identify a process that can be applied to actual patient management. As it is not always possible to measure all sICs and CK in actual medical practice, their importance must be evaluated and a process must be established to select these markers and to best utilize them. In this study, we implemented ML algorithms and comprehensively analyzed the prognostic prediction abilities of various clinical biomarkers, including sICs, CK, and infection biomarkers.

## 2 Materials and methods

### 2.1 Characteristics of study population

From May 2022 to September 2022, 148 specimens were collected from individuals (N = 100) diagnosed with SARS-COV-2 and had a history of hospitalization. The inclusion criteria were as follows: (i) serum sample within one week after admission, (ii) hospitalization, and (iii) detection of SARS-CoV-2 RNA from nasopharyngeal/throat swabs using real-time reverse transcription-polymerase chain reaction (RT-PCR). The CFX96 real-time PCR (Bio-Rad, Hercules, CA, USA) was used for detection of SARS-CoV-2 RNA. Pediatric patients and those with insufficient serum sample volumes were excluded. Among the 148 collected specimens, 100 were drawn during the first week after admission. These specimens were used for comparing non-survivors and survivors, as well as for evaluating differences between pneumonic and non-pneumonic conditions, conducting survival analysis, and ML analysis, including decision tree analysis. Additionally, values from the second week were measured in 48 patients, comprising of 42 survivors and 6 non-survivors. The trend of sICs and CK values between the first and second weeks was analyzed. This study was approved by the Institutional Review Board of Kangnam Sacred Heart Hospital at Hallym University. The requirement for informed consent was waived to maintain personal information anonymity (HKS. 2020-08-004-003).

### 2.2 Assessments of sIC regulators, CKs, and laboratory tests

Residual serum samples collected from the chemistry tests were aliquoted into microtubes and stored at -70°C for sIC and CK evaluation. The quantitative values of serum sICs and CKs were measured using a Luminex 200 Bio-Plex instrument (Bio-Rad, Hercules, CA, USA), following the manufacturer’s protocols. Measurements of sICs and CK were conducted using the methods described in a previous study (Jeong et al., 2022; Lee et al., 2022). Briefly, using a color-coded multiplexing method, Luminex 200 Bio-Plex simultaneously detects analytes through capture antibodies on beads, followed by fluorescence-based measurements for high-throughput immune profiling. In this study, we included 17 types of sICs and 16 types of CK that were previously measured in the literature (Lee et al., 2022). Laboratory data obtained on the same date as the measurement date for sIC and CK were retrospectively collected from medical records. It included procalcitonin (PCT), CRP, total WBC, absolute neutrophil, and lymphocyte counts. In addition, multidrug-resistant bacteria such as methicillin-resistant *S.aureus* (MRSA), carbapenem-resistant *Acinetobacter baumannii* (CRAB), and carbapenem-resistant *Enterobacteriaceae* (CRE) were investigated.

### 2.3 Statistical analysis

The Mann-Whitney U test was used to evaluate the statistically significant differences in survivors vs. non-survivors and pneumonic vs. non-pneumonic conditions groups. The area under the receiver-operating characteristic curve (AUC) of receiver operating characteristic (ROC) was used to evaluate the performance of each biomarker. The cutoff for the continuum value was calculated using the Youden index, and its sensitivity and specificity were evaluated. Pearson’s correlation coefficient was used to assess the linear relationship between various sICs, CKs, and infection markers (e.g., CRP, PCT). Cumulative overall survival curves for each sIC, CK, and laboratory biomarker were calculated using the Kaplan-Meier analysis and examined using the log-rank test. Cluster analysis was performed to comprehensively evaluate the factors affecting the prognosis of SARS-CoV-2 infected patients. K-means clustering algorithms were selected for clustering and the optimal number of clusters (K) was determined. A prognostic analysis was performed for each cluster. Paired samples from the 1^st^ and 2^nd^ weeks were compared using paired t-tests. Differences were considered statistically significant at P < 0.05. All statistical analyses were performed using MedCalc software version 12.0 (MedCalc Software, Mariakerke, Belgium).

### 2.4 ML

ML, based on a decision-tree algorithm, was used for survival and prognosis prediction. A decision tree is a data classification technique that is constructed in various ways as branch-like fragments. It consists of a root node, leaf node, and decision (internal) node, which indicate the node at the top, the class to be assigned to patients, and the tree corresponding to the features, respectively. The sICs, CK, and laboratory test data were all included as parameters and selected for survival and prognosis, prioritizing items that were statistically significant in the Mann-Whitney U analysis. Before training, patient identification information was removed from the raw data for preprocessing, and categorical variables were encoded using one-hot encoding to facilitate processing by the decision tree algorithm. The decision to split the dataset into 80% training and 20% testing was based on standard ML practices to balance learning capability and validation accuracy. Google Colaboratory, a Colabcloud-based service, was utilized (Hoyos-Rivera et al., 2006) to perform the ML operations. Training and prediction were performed using a decision tree classifier, the structure of the tree was visualized by drawing a plot, and information corresponding to the node was obtained. The decision tree classifier was configured with a maximum depth of 10 to prevent overfitting. The significance of each variable was analyzed through seaborn barplot and the accuracy and AUC of ROC curve were examined. In addition, to evaluate significance of the features for survival prediction and interpret the ML results, we utilized the SHapley Additive exPlanations (SHAP). SHAP values were calculated to quantify the contribution of each feature to the prediction outcome, providing a clearer picture of model decisions. This allowed us to identify which features were most influential and how they interacted to affect survival predictions. The code utilized in this study can be accessed online (https://github.com/Nurilee822/sICsandCK_COVID19).

## 3 Results

### 3.1 Patients

Of the 100 patients enrolled in the study, 19 died and 81 survived. Among the 100 patients, 49 were diagnosed with pneumonia and the remaining 51 patients showed mild symptoms. The average age was 82.0 and 73.0 years for non-survivors and survivors, respectively, and 80.0 and 72.0 for pneumonic and non-pneumonic conditions patients, respectively (**Table 1**). Fourteen patients had cancer, 50 had hypertension, 32 had diabetes mellitus (DM), 16 had cardiovascular disease, 36 had other diseases, and 20 had no relevant medical history. CRAB, CRE, and MRSA infections were present in six, one, and six patients, respectively. No statistically significant differences were exhibited in age, sex, underlying disease, and bacterial infections, such as CRAB, CRE, and MRSA between the non-survivors and survivors. In patients with pneumonia, no statistically significant disparity was observed in age or sex between the pneumonic and non-pneumonic conditions patients. Among the underlying diseases, cardiovascular diseases and CRAB bacterial infections were statistically noticeably increased in the pneumonic patients compared to those in the non-pneumonic conditions patients. WBC count, N-to-L ratio, PCT, and CRP were statistically significantly increased in the non-survivors and pneumonic patient groups compared to the survivors and non-pneumonic conditions patients.

**Table 1:** Demographics and characteristics of patients with SARS-CoV-2 infection.

Variables	Death			Pneumonia		
	Survivors (N = 81)	Non-survivors (N = 19)	*P* ^†^	Non-pneumonic (N = 51)	Pneumonic (N = 49)	*P*
Age, year	73.0 (60.0 – 83.5)	82.0 (72.3 – 84.8)	0.1218	72.0 (58.3 – 84.5)	80.0 (68.5 – 84.3)	0.1248
Gender (Male: Female)	39:42	11:8	0.4467	23:28	27:22	0.3196
Underlying disease (%) Cancer Hypertension Diabetes mellitus Cardiovascular disease Others^*^ None	12 (14.8%) 40 (49.4%) 27 (33.3%) 11 (13.6%) 29 (35.8%) 18 (22.2%)	2 (10.5%) 10 (52.6%) 5 (26.3%) 5 (26.3%) 7 (36.8%) 2 (10.5%)	0.6295 0.7998 0.5571 0.1751 0.9326 0.2537	5 (9.8%) 27 (52.9%) 17 (33.3%) 4 (7.8%) 19 (37.3%) 14 (27.5%)	9 (18.4%) 23 (46.9%) 15 (30.6%) 12 (24.5%) 17 (34.7%) 6 (12.2%)	0.2196 0.5504 0.7717 0.0239 0.7907 0.0586
CRAB (Yes: No)	4:77	2:17	0.3584	0:51	6:43	0.0117
CRE (Yes: No)	0:81	1:18	0.1900	0:51	1:48	0.3076
MRSA (Yes: No)	4:77	2:17	0.3584	2:49	4:45	0.3743
White blood cell count (× 10^9^/L)	5.92 (4.62 – 8.75)	11.01 (7.14 – 17.77)	0.0007	5.17 (4.31 – 7.84)	8.02 (6.22 – 13.2)	0.0007
Neutrophil to lymphocyte ratio	5.02 (2.29 – 9.19)	21.13 (10.42 – 32.14)	0.0002	3.33 (1.95 – 7.68)	11.0 (5.58 – 20.4)	<0.0001
Procalcitonin (ng/mL)	0.13 (0.07 – 0.23)	3.66 (0.18 – 17.83)	0.0003	0.12 (0.04 – 0.21)	0.22 (0.11 – 2.12)	0.0047
C-reactive protein (mg/dL)	30.7 (7.6 – 52.9)	69.8 (45.4 – 246.7)	0.0001	17.6 (3.40 – 46.7)	49.2 (33.6 – 94.5)	<0.0001

Values are presented as median (IQR).

^*^Others include dyslipidemia (N=15), chronic kidney disease (N=15), pulmonary tuberculosis (N=6), hepatic disease (N=2), and epilepsy (N=1).

^†^The p-values indicate the statistical significance of the difference between survival and non-survival in the first and second weeks.

CRE, carbapenem-resistant *Enterobacteriaceae*; CRAB, carbapenem-resistant *Acinetobacter baumannii*; MRSA, methicillin-resistant *S.aureus*.

### 3.2 sIC regulators and CKs

**Figure 1:**
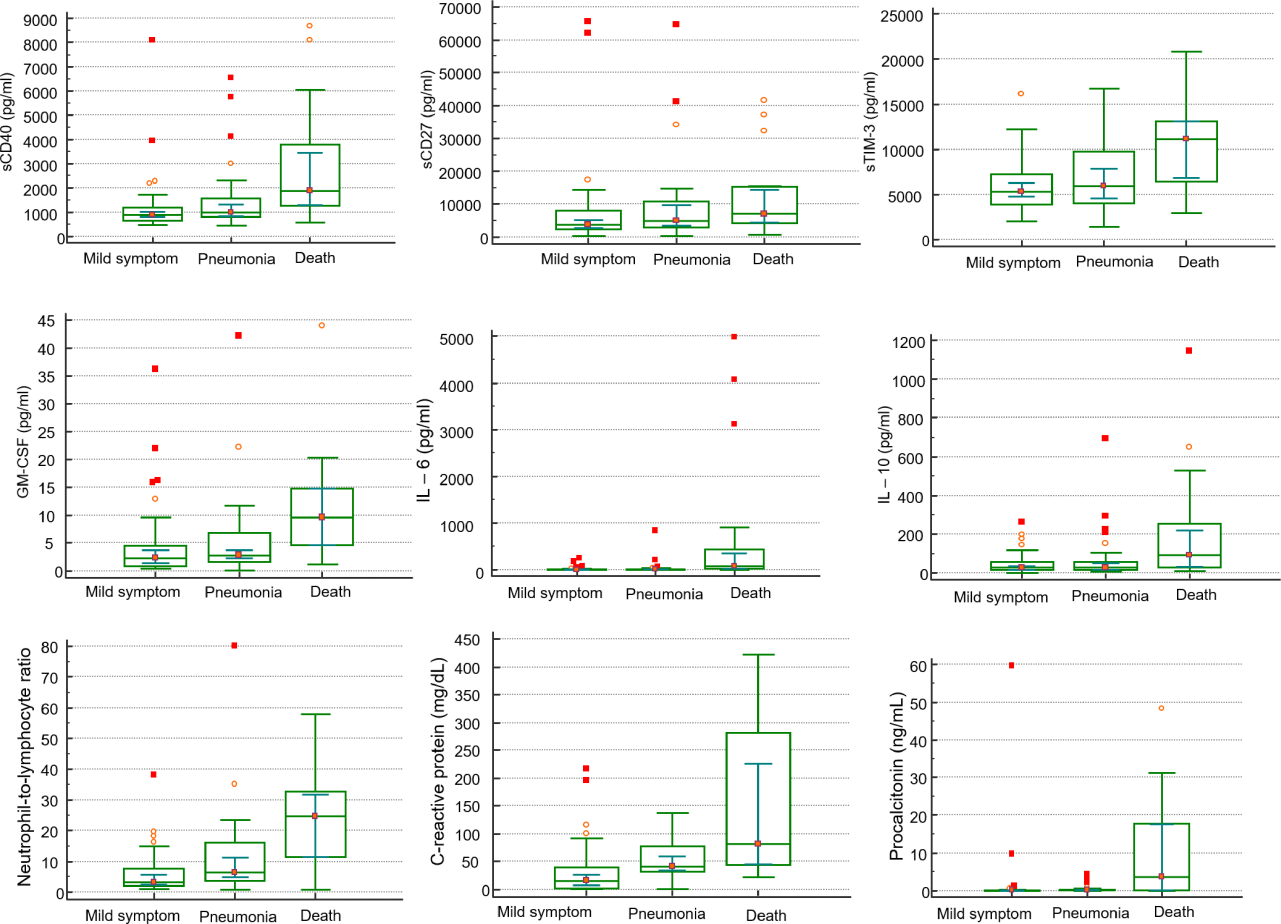
Comparison of clinical markers, sICs, CKs in mild symptom (N=48), pneumonia (N=33), and death (N=19) in patients infected with SARS-CoV-2 by Mann-Whitney U test.

Among sICs, soluble clusters of differentiation (sCD)27, sCD40, soluble herpes virus entry mediator (sHVEM), soluble T-cell immunoglobulin and mucin-domain containing-3 (sTIM-3), and soluble Toll-like receptor 2 (sTLR-2) were significantly increased in the non-survivors compared to those in the survivors. Soluble B-lymphocyte and T-lymphocyte attenuator (sBTLA) and soluble lymphocyte-activation gene 3 (sLAG-3) showed significantly lower values in the non-survivors compared to those in the survivors. Among CKs, chemokine CC motif ligand 2 (CCL2), granulocyte-macrophage colony-stimulating factor (GM-CSF), interleukin 6 (IL-6), IL-8, IL-10, IL-13, and tumor necrosis factor-α (TNF-α) were significantly increased in the non-survivors compared to those in the survivors. In the case of pneumonia, sBTLA and sLAG-3 among sICs were significantly lowered in the pneumonic patients compared to those in the non-pneumonic conditions patients, and sCD40 and sTIM-3 were statistically substantially increased in the pneumonic patients than those in the non-pneumonic conditions patients. CK, GM-CSF, and IL-6 levels were higher in pneumonic patients than in non-pneumonic conditions patients (**Table 2**, **Figure 1**). **Figure 1** includes the top three markers (existing inflammatory markers, sICs, and CK) used in this study. These markers are ranked based on their P-value, with inclusion only when the P-value was significant at <0.05.

**Table 2:** Levels of soluble type immune checkpoint regulators (sICs) and cytokines (CKs) in patients with SARS-CoV-2 infection .

Variables		Death			Pneumonia		
		Survivors (N = 81)	Non-survivors (N = 19)	*P* ^†^	Non-pneumonic (N = 51)	Pneumonic (N = 49)	*P*
sICs (pg/ml)	sBTLA	247.2 (153.9 – 372.2)	153.9 (96.7 – 245.6)	0.0247	247.2 (163.3 – 399.0)	191.5 (115.6 – 283.9)	0.0429
	sCD27	4231.8 (2385.2 – 9699.4)	7384.6 (4347.1 – 15410.6)	0.0112	3860.6 (2390.2 – 8295.3)	6379.3 (3557.3 – 11818.0)	0.054
	sCD28	2233.5 (1619.6 – 3041.7)	1873.8 (1632.3 – 3072.8)	0.8056	2211.7 (1632.3 – 2904.9)	2285.2 (1619.6 – 3241.3)	0.8496
	sCD40	919.1 (767.6 – 1276.6)	1950.3 (1303.6 – 5466.7)	0.0001	885.6 (691.5 – 1194.8)	1283.6 (837.2 – 2148.7)	0.0055
	sCD80/B7-1	48.8 (30.4 – 76.9)	34.5 (17.9 – 55.8)	0.1188	50.8 (33.4 – 81.5)	40.8 (26.9 – 63.3)	0.2467
	sCD86/B7-2	43.5 (26.6 – 72.9)	36.7 (18.9 – 70.7)	0.3891	43.5 (23.0 – 74.2)	36.7 (29.9 – 72.7)	0.7101
	sCTLA-4	6.35 (4.57 – 8.66)	7.25 (4.24 – 8.77)	0.6958	6.56 (5.41 – 9.16)	6.13 (4.13 – 8.60)	0.3929
	sGITR	61.4 (30.3 – 98.4)	43.1 (23.4 – 83.1)	0.4154	73.2 (31.1 – 98.6)	45.4 (27.0 – 92.6)	0.4598
	sGITRL	89.7 (42.8 – 148.2)	49.9 (21.4 – 98.8)	0.0621	87.6 (42.4 – 134.7)	81.6 (23.1 – 137.5)	0.4443
	sHVEM	4230.7 (3257.3 – 5818.1)	5846.8 (4121.1 – 12682.2)	0.0456	4099.0 (3219.8 – 5858.0)	4768.5 (3609.9 – 6799.7)	0.1937
	sICOS	513.1 (315.0 – 867.8)	294.6 (164.2 – 718.3)	0.0548	577.2 (316.9 – 911.6)	383.0 (227.0 – 829.0)	0.1081
	sLAG-3	49161.3 (37125.3 – 69626.4)	36111.1 (19124.2 – 59842.4)	0.0402	55818.7 (36559.0 – 69643.6)	43475.3 (27070.0 – 60494.2)	0.0474
	sPD-1	356.3 (260.2 – 492.8)	389.7 (316.6 – 663.8)	0.3449	373.9 (276.7 – 531.5)	340.6 (268.0 – 478.7)	0.6391
	sPD-L1	52.3 (37.8 – 76.0)	54.5 (36.9 – 68.4)	0.9930	59.2 (41.6 – 80.0)	50.9 (35.2 – 68.2)	0.1996
	sPD-L2	13443.0 (10609.1 – 14858.1)	12368.7 (10861.3 – 15605.3)	0.8460	13800.6 (10906.9 – 16015.4)	12354.4 (9826.5 – 14105.5)	0.0692
	sTIM-3	5637.0 (3977.6 – 8142.7)	11749.4 (6674.2 – 14169.8)	0.0002	5301.3 (3791.2 – 7850.8)	7328.6 (4653.7 – 10976.7)	0.0142
	sTLR-2	944.4 (675.6 – 1208.9)	1121.7 (907.1 – 1801.9)	0.0251	992.9 (622.8 – 1250.4)	1015.3 (819.9 = 1378.8)	0.3868
CKs (pg/ml)	CCL2	128.0 (84.0 – 235.8)	298.5 (112.9 – 556.9)	0.0163	123.8 (83.4 – 248.1)	157.7 (101.9 – 324.5)	0.2509
	CCL3	6.06 (3.15 – 11.3)	5.53 (3.0 – 26.5)	0.5683	4.78 (2.30 – 9.17)	8.38 (4.46 – 16.13)	0.3760
	CCL4	45.2 (28.6 – 72.9)	84.6 (34.1 – 155.7)	0.0630	42.7 (25.5 – 60.7)	64.8 (31.4 – 113.5)	0.0582
	CXCL10	51.6 (27.2 – 98.3)	83.9 (28.8 – 189.1)	0.1189	56.8 (30.6 – 105.8)	50.9 (26.7 – 131.0)	0.9588
	GM-CSF	2.69 (1.13 – 4.63)	9.66 (4.63 – 14.84)	0.0013	2.36 (0.94 – 4.63)	3.82 (2.32 – 11.12)	0.0354
	IFN-α	0.85 (0.49 – 2.86)	2.05 (1.37 – 4.02)	0.1766	0.93 (0.63 – 2.28)	1.38 (0.52 – 4.05)	0.7565
	IFN-γ	1.48 (0.77 – 4.32)	1.02 (0.87 – 1.46)	0.3707	1.60 (0.80 – 7.53)	1.09 (0.83 – 2.84)	0.1259
	IL-10	27.6 (12.1 – 59.7)	83.0 (28.7 – 233.0)	0.0023	29.0 (12.1 – 68.3)	38.7 (17.7 – 117.3)	0.0789
	IL-12p70	3.06 (2.23 – 3.47)	3.47 (2.43 – 7.60)	0.1074	3.06 (2.43 – 3.47)	3.06 (2.18 – 4.12)	0.8348
	IL-13	15.8 (12.7 – 19.8)	22.4 (13.5 – 24.8)	0.0199	13.5 (10.0 – 21.5)	16.9 (13.5 – 22.1)	0.1967
	IL-1α	2.09 (1.42 – 2.97)	2.96 (1.63 – 4.33)	0.0697	1.97 (1.32 – 3.24)	2.31 (1.63 – 3.36)	0.1949
	IL-1β	0.53 (0.29 – 0.91)	0.69 (0.34 – 1.47)	0.3495	0.53 (0.34 – 0.91)	0.53 (0.32 – 0.96)	0.7892
	IL-4	0.12 (0.06 – 0.20)	0.22 (0.06 – 0.30)	0.4442	0.12 (0.07 – 0.20)	0.12 (0.06 – 0.29)	0.5675
	IL-6	8.28 (1.82 – 16.9)	72.8 (23.8 – 392.5)	<0.0001	6.32 (1.73 – 18.6)	16.4 (9.25 – 67.8)	0.0042
	IL-8	11.8 (7.85 – 20.1)	24.8 (12.7 – 187.5)	0.0033	12.0 (7.63 – 22.6)	16.4 (9.51 – 28.6)	0.1410
	TNF-α	5.28 (2.85 – 8.56)	8.89 (5.31 – 16.0)	0.0082	5.31 (2.85 – 8.33)	7.62 (3.74 – 10.8)	0.0346

Values are presented as median (IQR).

^*^Total number of patients available for measurement of cytokine levels.

^†^The p-values indicate the statistical significance of the difference between survival and non-survival in the first and second weeks.

BTLA, B- and T-lymphocyte attenuator; CD, cluster of differentiation; CTLA-4, cytotoxic T-lymphocyte-associated protein 4; GITR, glucocorticoid-induced TNFR-related protein; GITRL, ligand for receptor TNFRSF18/AITR/GITR; HVEM, herpes virus entry mediator; ICOS, inducible T-cell costimulator; LAG-3, lymphocyte-activation gene 3; PD-1, programmed cell death protein 1; PD-L1, programmed death-ligand 1; TIM-3, T-cell immunoglobulin and mucin-domain containing-3; TLR-2, Toll-like receptor 2; CCL, chemokine CC motif ligand; CXCL, C-X-C motif chemokine ligand; GM-CSF, Granulocyte-macrophage colony-stimulating factor; IFN, Interferon; IL, Interleukin; TNF, tumor necrosis factor.

In correlation analysis showed that CRP has a positive correlation with sCD40 (r^2^ = 0.414, P<0.0001), sHVEM (r^2^ = 0.359, P=0.0002), TIM-3 (r^2^ = 0.446, P<0.0004), while showing a significant negative correlation with sLAG-3 (r^2^=-0.367, P=0.0002). PCT also shows a statistically significant positive correlation with sCD40 (r^2^ = 0.370, P=0.0014), sHVEM (r^2^ = 0.318, P=0.0064), and sTIM-3 (r^2^ = 0.397, P=0.0006). In case of CK, 11 CKs including CCL2, CCL4, GM-CSF, IL-1, IL-4, IL-6, IL-9, IL-10, IL-12, and TNF-a showed moderate correlation with CRP, and/or PCT (**Supplementary Table 1**).

### 3.3 Survival analysis

**Figure 2:**
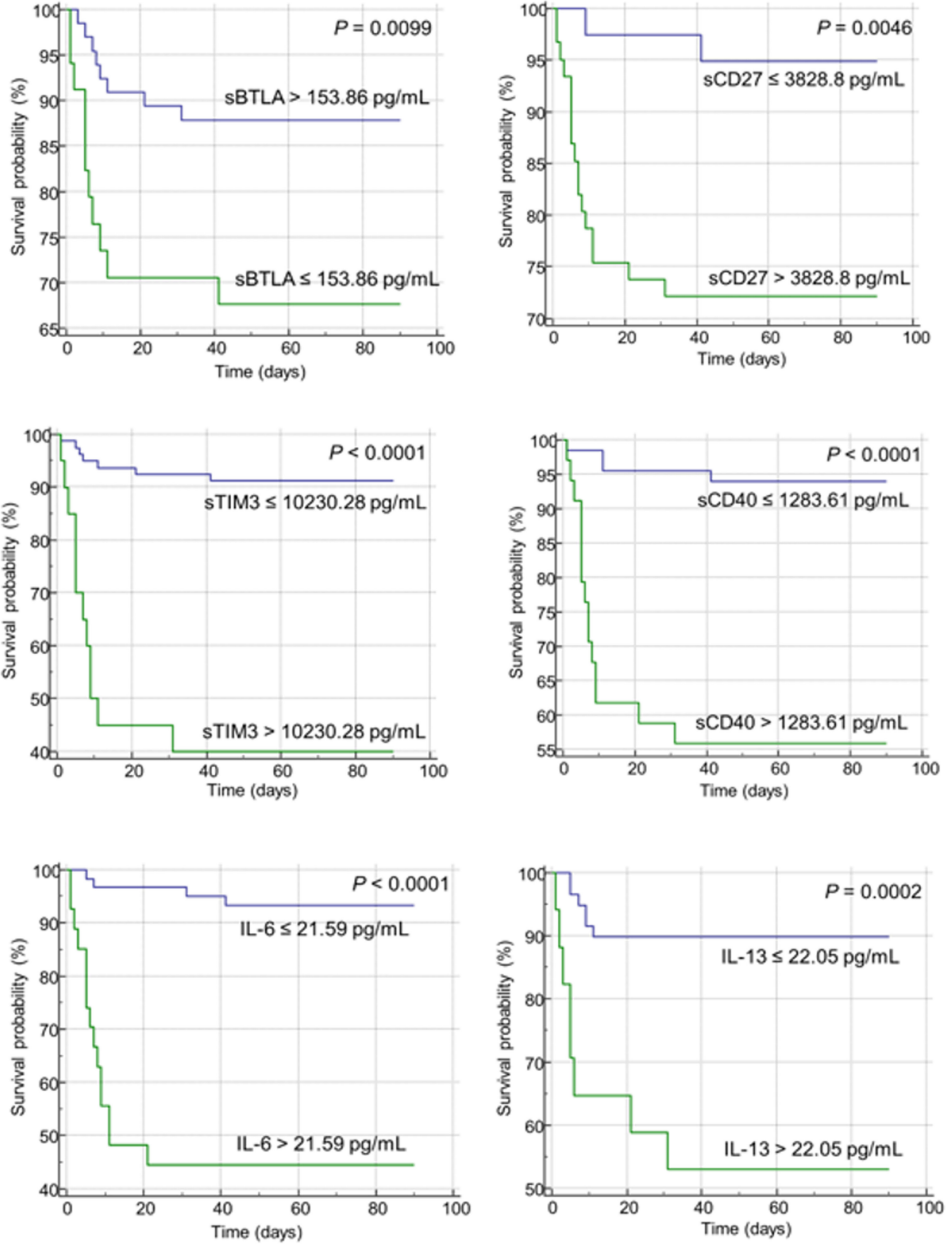
Kaplan-Meier curves of soluble immune checkpoints (sICs) and cytokines for the overall survival in patients infected with SARS-CoV-2. The green and blue lines represent patient groups divided based on the Youden index for each item. The green line represents patients with an unfavorable prognosis in the Kaplan-Meier analysis, while the blue line indicates a patient group showing a relatively favorable prognosis. Increased level of sICs, including sCD27, sCD40, and sTIM-3, were significantly associated with poor prognosis in the log-rank test. Decreased level of sBTLA was correlated with poor prognosis. In case of cytokines, increased levels of IL-6 and IL-13 were associated with a poor prognosis.

The AUC, cutoff value, sensitivity, and specificity of each indicator to discriminate non-survivors in patients with COVID-19 infection are presented in **Table 3**. The area under the curve for each indicator ranged from 0.652 to 0.844. IL-6 showed the highest AUC value (0.844, 95% CI = 0.751 – 0.913), with a sensitivity of 78.9% and specificity of 82.4% at a cut off 21.59 set by the Youden index (**Table 3**). When Kaplan-Meier 90-d survival analysis was performed, patients with factors as follows showed poor prognosis with statistical significance (**Figure 2**, **Supplementary Figure 1**): PCT over 0.25 ng/mL, CRP over 41.0 mg/dL, WBC over 6.32 × 10^9^/L, N-to-L ratio over 18.97, sBTLA below 153.86 pg/mL, sCD27 over 3828.8 pg/mL, sCD40 over 1283.61 pg/mL, sTIM-3 over 10230.28 pg/mL, sLAG-3 under 43475.3 pg/mL, sTLR-2 over 857.67 pg/mL, CCL-2 over 297.51 pg/mL, GM-CSF over 5.28 pg/mL, IL-6 over 21.59 pg/mL, IL-10 over 69.1 pg/mL, IL-13 over 22.05 pg/mL, and TNF-α over 7.65 pg/mL. **Figure 2** includes items that demonstrated significance in the Kaplan-Meier analysis. The most important features identified in this analysis were further scrutinized in the decision tree analysis.

**Table 3:** Area under the Receiver Operating Characteristic (ROC) curve, 95% confidence interval, cut-off point, sensitivity, specificity, p-value to discriminate non-survival in patients with COVID-19 infection.

Indicator	Area under the ROC curve (95% CI)	Cut-off point	Sensitivity (%)	Specificity (%)	*P*-value
White blood cell count (× 10^9^/L)	0.750 (0.653 – 0.831)	6.32	89.5	51.9	<0.001
Neutrophil to lymphocyte ratio	0.822 (0.733 – 0.891)	18.97	63.2	91.4	<0.001
Procalcitonin (ng/mL)	0.797 (0.686 – 0.883)	0.25	68.8	78.6	<0.001
C-reactive protein (mg/dL)	0.791 (0.698 – 0.866)	41.0	84.2	66.7	<0.001
sBTLA (pg/ml)	0.666 (0.564 – 0.758)	153.86	57.9	72.5	0.015
sCD27 (pg/ml)	0.687 (0.587 – 0.776)	3828.8	89.5	45.7	0.005
sCD40 (pg/ml)	0.787 (0.694 – 0.863)	1283.61	78.9	76.5	<0.001
sLAG-3 (pg/ml)	0.652 (0.550 – 0.744)	43475.3	68.4	64.2	0.0342
sTIM-3 (pg/ml)	0.776 (0.682 – 0.854)	10230.28	63.2	90.1	<0.001
sTLR-2 (pg/ml)	0.666 (0.564 – 0.757)	857.67	89.5	43.2	0.016
CCL2 (pg/ml)	0.678 (0.577 – 0.768)	297.51	52.6	88.9	0.033
GM-CSF (pg/ml)	0.767 (0.660 – 0.854)	5.28	73.3	80.3	<0.001
IL-6 (pg/ml)	0.844 (0.751 – 0.913)	21.59	78.9	82.4	<0.001
IL-8 (pg/ml)	0.717 (0.619 – 0.803)	23.88	52.6	84.0	0.003
IL-10 (pg/ml)	0.726 (0.627 – 0.811)	69.1	57.1	80.0	<0.001
IL-13 (pg/ml)	0.699 (0.583 – 0.799)	22.05	57.1	85.5	0.023
TNF-α (pg/ml)	0.704 (0.595 – 0.799)	7.65	61.1	72.7	0.007

BTLA, B- and T-lymphocyte attenuator; CD, cluster of differentiation; LAG-3, lymphocyte activation gene 3; TIM-3, T-cell immunoglobulin and mucin-domain containing-3; TLR-2, Toll-like receptor 2; CCL, chemokine CC motif ligand; CXCL, C-X-C motif chemokine ligand; GM-CSF, Granulocyte-macrophage colony-stimulating factor; IL, Interleukin; TNF, tumor necrosis factor.

K-means clustering was conducted and the optimal number was determined to be three. Among clusters 1, 2, and 3, cluster 2 exhibited the poorest prognosis, whereas cluster 3 showed the most favorable prognosis. Cluster 2 showed statistically significant final cluster centers with PCT 14.7 ng/mL, CRP 161.52 mg/dL, N-to-L ratio 43.6, sCD27 37248.92 pg/mL, sTIM-3 17070.16 pg/mL, sCD40 6520.67 pg/mL, GM-CSF 14.74 pg/mL, IL-6 1232.37 pg/mL, and TNF-α 17.32 pg/mL and these values corresponded to the highest among the three clusters (**Supplementary Figure 2**).

### 3.4 ML application for predicting prognosis

**Figure 3:**
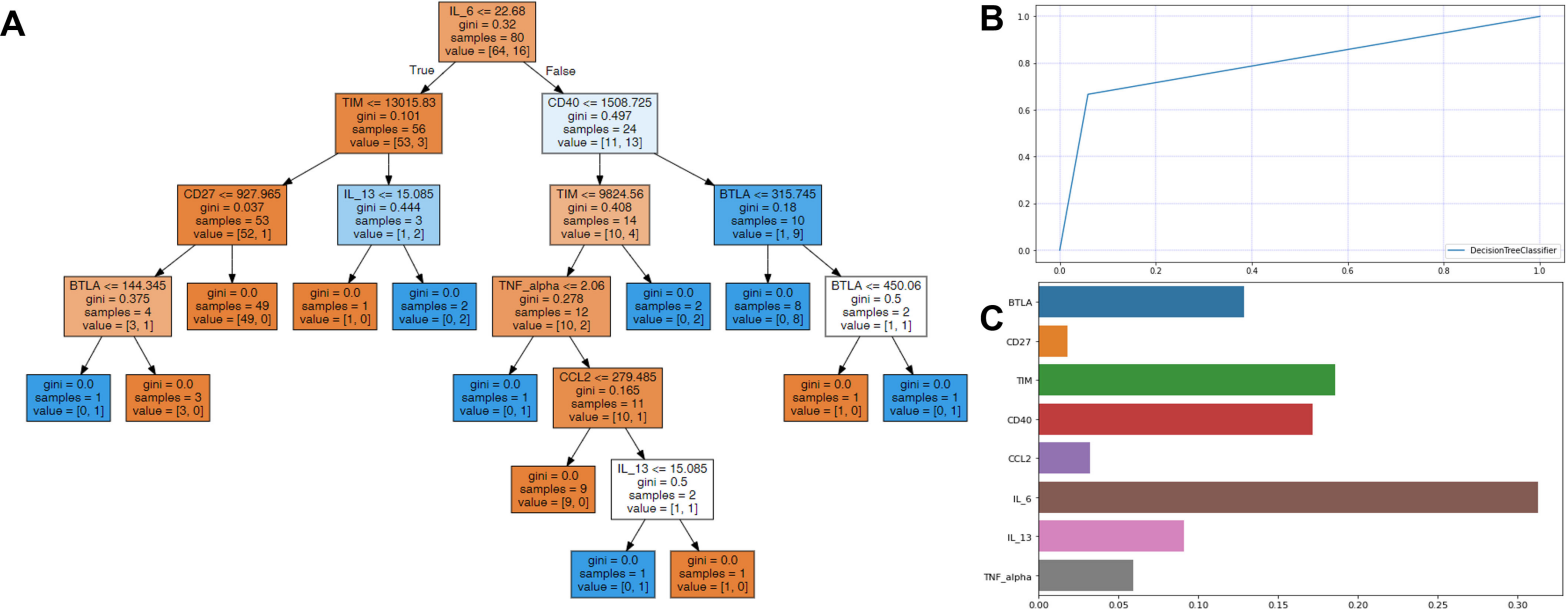
Decision tree analysis for predicting the survival in patients infected with SARS-CoV-2. **(A)** In each node, gini represented the quantified value of purity in the node, and a sample is the number of patients. The color demonstrate represents the class that encompasses the majority of samples within each node, and orange color for survivors and blue for non-survivors. In the prediction processing, at the root node, the patients were divided into two groups, with IL-6 value ≤22.68 or not. Then, the divided patients were classified by the second layer leaf node with sTIM-3 or sCD40. **(B)** The survival prediction AUC of the decision tree was 0.803 and **(C)** IL-6, sTIM-3, sCD40, sBTLA, and IL-13 were shown to be most important features among sICs and CKs.

**Figure 4:**
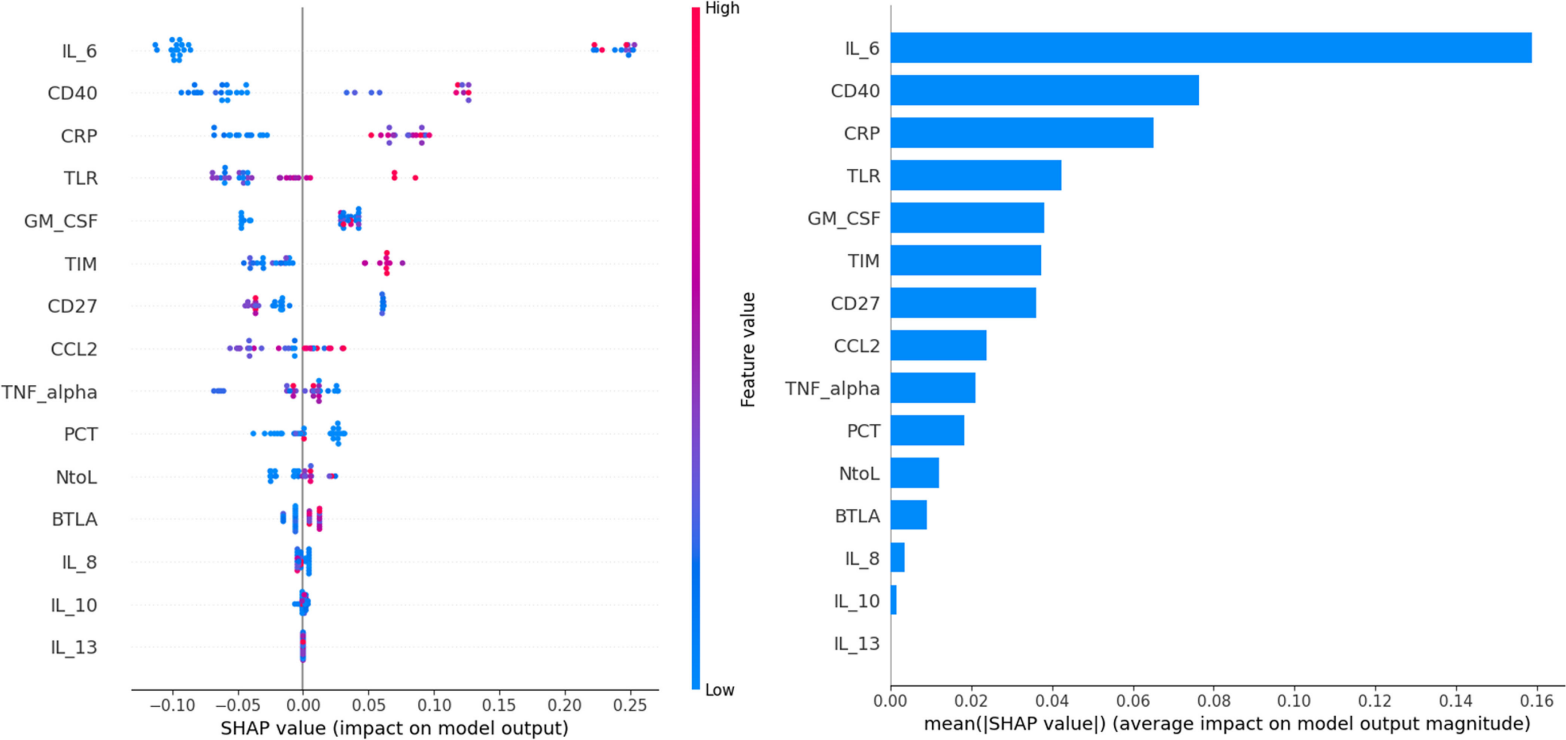
The top 15 risk factors evaluated by the average absolute SHAP value. The SHAP value (x-axis) is a unified index that responds to the influence of a mortality in the model, and the feature ranking (y-axis) indicates the importance of the predictive model. All patients’ attributes for the outcome are plotted in rows, as colored dots, where red and blue dots represent the high risk values and low risk values, respectively.

The enrolled individuals diagnosed with SARS-CoV-2 were analyzed utilizing decision tree analysis. Decision tree analysis was performed based on sICs and CKs, with significantly different levels between the survivors and non-survivors. The decision tree and feature importance are shown in **Figure 3**. According to these rules, IL-6 was the root node with the largest information gain when applying the built-in sklearn analysis. In the second layer of the leaf nodes, the sTIM-3 and sCD40 values were the classification standards. The conditions where IL-6 exceeded 22.68 and both sCD40 surpassed 1508.725 and sBTLA was below 315.745, were classified as non-survivors. Additionally, when the value of IL-6 was 22.68 or below, it was classified as survivors based on sTIM-3 being below 13015.83 and sCD27 below 927.965. The survival prediction AUC of the decision tree was 0.803, with an accuracy of 0.9. When the significance of each variable in the decision tree was analyzed using a Seaborn bar plot, IL-6, sTIM-3, sCD40, and sBTLA had a high impact on the model (**Figure 3**). Furthermore, we used the SHAP method to identify the most important features of the enrolled patients and visually explain how these variables affected the mortality rate. The top 15 most important features of this study and the SHAP values for each biomarker in the model output are presented in **Figure 4**. IL-6, sCD40, and CRP are the top three important biomarkers associated with a higher predicted 90-d mortality in patients diagnosed with SARS-CoV-2. In addition, sTLR-2, GM-CSF, TIM-3, CD27, CCL-2, TNF-α, PCT, N-to-L ratio, sBTLA, IL-8, IL-10, and IL-13 have been selected as significant top 15 features (**Figure 4**).

### 3.5 Comparing paired samples between 1^st^ and 2^nd^ weeks with SARS-CoV-2 infection

**Figure 5:**
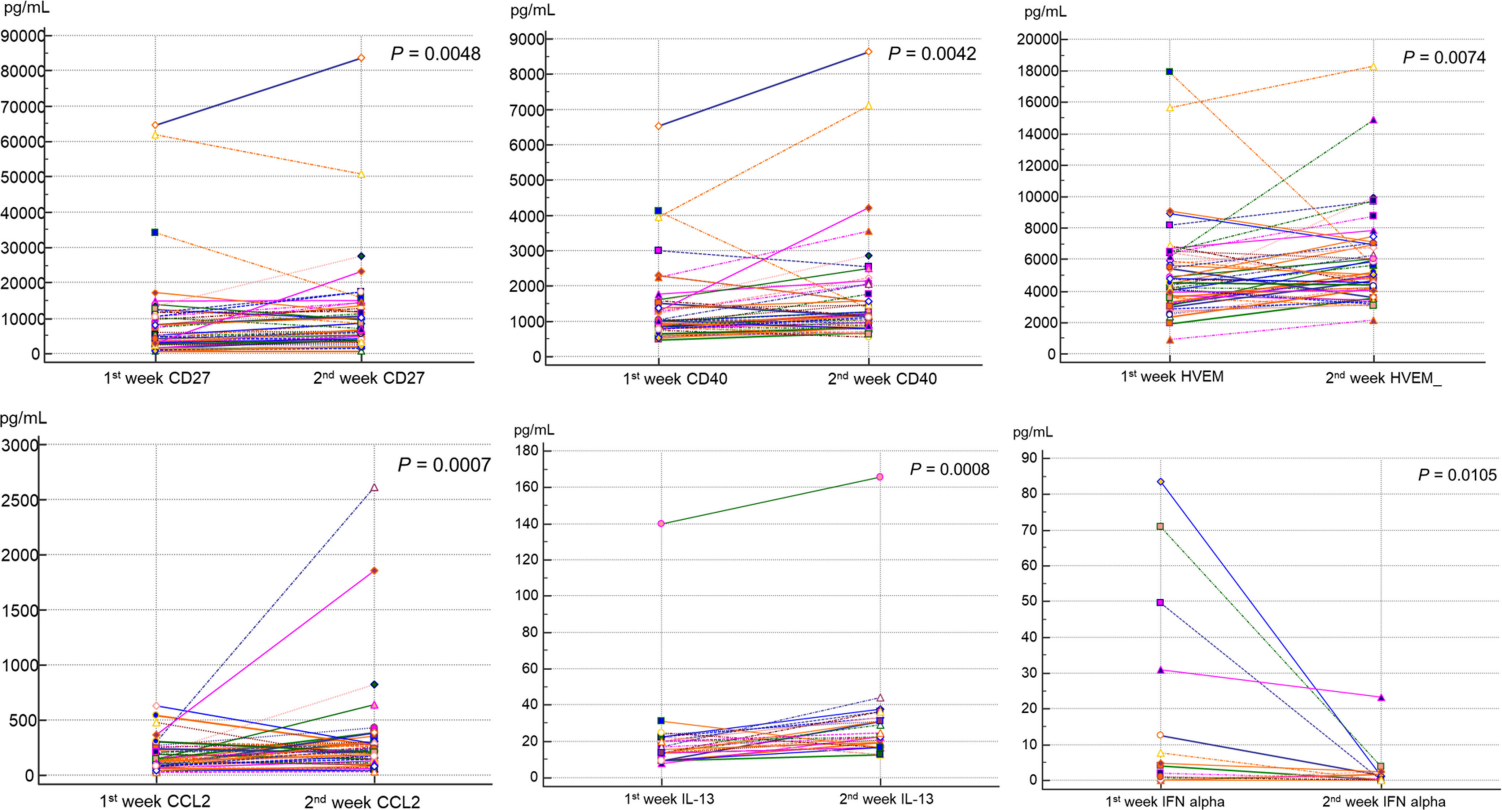
Comparison of sIC and CK levels between the first and second weeks after SARS-CoV-2 infection by paired Wilcoxon-rank test. sCD27 showed median level of 4912.4 pg/mL in first week and increased to 6685.6 pg/mL in the second week (P = 0.0048). sCD40 showed median level of 986.7 pg/mL and 1181.6 pg/mL, in first and second weeks respectively (P = 0.0042). sHVEM (P = 0.0074), CCL2 (P = 0.0007), and IL-13 (P = 0.0008) also displayed significantly increased values at second week compared to those in the first week. IFN-α showed a significantly lower value in the second week (0.86 pg/mL) compared to that in the first week (4.40 pg/mL, P = 0.0105).

Among all patients, the sICs and CK values in the 1^st^ and 2^nd^ weeks were compared pairwise in 48 patients (**Table 4**; **Figure 5**). Among the sICs, the sCD27, sCD28, sCD40, sHVEM, sLAG-3, and sTIM-3 levels were significantly higher in the 2^nd^ week than those in the initial week. Among the CKs, CCL2, CCL3, CCL4, IL-8, and IL-13 showed statistically significant increases in the 2^nd^ week in comparison with those in the 1^st^ week. On the other hand, IFN-α significantly decreased in the 2^nd^ week than that in the 1^st^ week. IL-6 did not show a significant statistical variation in values between the 1^st^ and 2^nd^ weeks, and it displayed increased values in the initial week (12.4 pg/mL, 95% CI: 5.01–32.0) as well as in the 2^nd^ week (15.4 pg/mL, 95% CI: 4.95–62.7). In addition, IL-10 and GM-CSF levels did not show statistical significance between the 1^st^ and 2^nd^ week. All sIC and CK data exhibiting a statistically significant difference between the values in the two weeks are included in the supplemental data (**Supplementary Figure 3**).

**Table 4:** Comparison of differences in median values according to measurement time of paired samples using Wilcoxon-rank test.

Variables		Paired samples		
		1^st^ week (N = 48)	2^nd^ week (N = 48)	*P*
sICs (pg/ml)	sBTLA	228.7 (138.4 – 352.6)	217.4 (170.4 – 331.8)	0.0845
	sCD27	4912.4 (2812.8 – 10300.7)	6685.6 (3653.4 – 12317.0)	0.0048
	sCD28	1989.0 (1556.5 – 3198.1)	2702.3 (2110.5 – 3927.3)	0.0001
	sCD40	986.7 (814.9 – 1454.0)	1181.6 (825.0 – 1674.6)	0.0042
	sCD80/B7-1	45.2 (25.9 – 76.5)	44.3 (28.4 – 92.4)	0.3051
	sCD86/B7-2	67.9 (32.5 – 92.7)	50.4 (20.1 – 92.3)	0.5538
	sCTLA-4	8.14 (5.96 – 10.3)	3.91 (2.55 – 6.56)	0.1250
	sGITR	54.2 (27.0 – 89.5)	63.3 (47.6 – 89.9)	0.2078
	sGITRL	72.4 (28.3 – 163.8)	86.7 (46.1 – 145.2)	0.0528
	sHVEM	4337.1 (3326.9 – 6123.6)	4958.6 (4166.0 – 6873.9)	0.0074
	sICOS	555.8 (316.9 – 932.2)	528.3 (334.0 – 847.0)	0.4157
	sLAG-3	44645.1 (28294.2 – 64357.4)	59051.5 (41215.4 – 77281.5)	0.0042
	sPD-1	323.0 (241.4 – 479.7)	375.5 (269.9 – 497.0)	0.5797
	sPD-L1	50.0 (42.8 – 62.0)	51.9 (37.5 – 72.1)	0.2146
	sPD-L2	12358.0 (9977.8 – 14492.0)	13138.3 (10454.2 – 14641.9)	0.6666
	sTIM-3	5834.6 (4043.1 – 9295.0)	7469.1 (5029.4 – 9410.2)	0.0001
	sTLR-2	917.3 (670.9 – 1229.6)	837.2 (619.3 – 1161.0)	0.0131
CKs (pg/ml)	CCL2	129.8 (80.9 – 216.8)	227.9 (132.3 – 296.7)	0.0007
	CCL3	16.0 (4.03 – 33.0)	44.4 (26.5 – 58.0)	0.0002
	CCL4	34.2 (26.1 – 91.4)	113.7 (37.2 – 380.5)	0.1250
	CXCL10	68.1 (25.0 – 108.9)	56.1 (27.7- 105.1)	0.1142
	GM-CSF	3.09 (1.38 – 6.36)	4.17 (1.85 – 6.17)	0.7802
	IFN-α	4.40 (0.64 – 30.9)	0.86 (0.09 – 2.46)	0.0105
	IFN-γ	1.44 (0.67 – 4.00)	2.09 (1.20 – 5.12)	0.5570
	IL-10	32.8 (15.5 – 81.8)	26.8 (11.8 – 52.4)	0.1756
	IL-12p70	3.06 (2.43 – 3.47)	3.06 (1.26 – 4.12)	0.7350
	IL-13	16.9 (13.5 – 20.5)	24.5 (19.4 – 36.3)	0.0008
	IL-1α	2.30 (1.63 – 2.99)	2.46 (1.32 – 3.82)	0.0723
	IL-1β	0.53 (0.36 – 0.91)	0.53 (0.20 – 1.62)	0.2134
	IL-4	0.15 (0.06 – 0.24)	0.29 (0.11 – 0.47)	0.0987
	IL-6	12.4 (5.01 – 32.0)	15.4 (4.95 – 62.7)	0.0951
	IL-8	14.4 (8.90 – 22.3)	20.4 (13.1 – 28.1)	0.0041
	TNF-α	6.26 (2.85 – 9.21)	5.45 (3.67 – 10.8)	0.2865

BTLA, B- and T-lymphocyte attenuator; CD, cluster of differentiation; CTLA-4, cytotoxic T-lymphocyte-associated protein 4; GITR, glucocorticoid-induced TNFR-related protein; GITRL, ligand for receptor TNFRSF18/AITR/GITR; HVEM, herpes virus entry mediator; ICOS, inducible T-cell costimulator; LAG-3, lymphocyte-activation gene 3; PD-1, programmed cell death protein 1; PD-L1, programmed death-ligand 1; TIM-3, T-cell immunoglobulin and mucin-domain containing-3; TLR-2, Toll-like receptor 2; CCL, chemokine CC motif ligand; CXCL, C-X-C motif chemokine ligand; GM-CSF, Granulocyte-macrophage colony-stimulating factor; IFN, Interferon; IL, Interleukin; TNF, tumor necrosis factor.

## 4 Discussion

In this study, several sICs and CKs were measured among those with SARS-CoV-2 diagnosed patients and the values in the survivors and non-survivors, and pneumonic and non-pneumonic conditions groups were compared. In addition, the clinical value of sICs and CKs was analyzed as additional markers useful for predicting prognosis along with existing clinical indicators. In addition to basic statistical analysis, ML analysis was used to screen factors of high importance. Assessing the significance of different biomarkers in predicting prognosis and assigning priority orders has considerable clinical relevance. During the extended period of SARS-CoV-2 infection, various studies have analyzed the prognostic prediction of several infection factors, including CRP, PCT, and CKs. However, few studies have comprehensively analyzed these markers and explored their relationships. Hence, in this study, the simultaneous measurement of various infection markers, sICs, and CKs was conducted, and the significance of each marker in predicting the prognosis of individuals affected by SARS-CoV-2 was analyzed.

WBC, N-to-L, PCT, and CRP, which have previously been noted to be significant in predicting the prognosis of patients diagnosed to SARS-CoV-2 (Ponti et al., 2020; Bedel and Korkut, 2021; Agarwal, 2022), were significantly higher in the non-survivors and pneumonic patients compared to those in the survivors and non-pneumonic conditions patients in this study. Among the sICs, sCD27, sCD40, sHVEM, and sTIM-3 showed considerably elevated values in the non-survivors than those in the survivors and sLAG-3 showed markedly reduced values in the non-survivors compared to those in the survivors. In case of CKs, CCL-2, GM-CSF, IL-6, IL-8, IL-10, IL-13, and TNF-alpha were statistically noticeably raised in the non-survivors compared to those in the survivors. All of these factors, except sLAG-3, showed worse prognosis in patients with elevated values than in those with decreased values in the Kaplan-Meier analysis. In the case of sLAG-3, the prognosis was worse in patients with lower values than in those with higher values. sICs play an important role in immune-mediated infection control by inducing immune responses that activate effector functions across diverse immune cells during viral infections (Bullock, 2017; Remedios et al., 2019; Deng et al., 2021; Joseph et al., 2021). Among the sICs, CD27 and CD40, which were identified as factors with high prognostic power through ML analysis in this study, play important roles in promoting the survival and effector functions of natural killer/T cells in viral infection, IFN-I response, and inhibition of its signaling (Yao et al., 2016; Bullock, 2017; Remedios et al., 2019; Deng et al., 2021). CD27 supports the initial activation and proliferation of T-cells, effectively enhancing their cytolytic and cytokine functions of cells (Bullock, 2017). The increase in sCD27 has been reported to play a crucial role in augmenting the appropriate immune cell response while inhibiting T cell proliferation (Penatzer et al., 2022). CD40, expressed on CD4+ T cells, also plays a significant role in stimulating T cells and promoting the formation of germinal centers and antibody class switching. Additionally, CD40 is associated with various cellular immune processes, such as T and B cell activation and apoptosis. It has been documented to function as a proinflammatory mediator in inflammatory diseases and may serve as an indicator for sepsis mortality (de Freitas E Silva and von Stebut, 2021; Liu et al., 2022). While we measured CD40, we could not include CD40L due to the unavailability of a commercial kit. CD40L, expressed on activated T cells, interacts with CD40 on various cell types, playing a crucial role in immune responses. The soluble form of CD40L (sCD40L) can exacerbate inflammation and contribute to the cytokine storm seen in severe SARS-CoV-2 infection (Hashem et al., 2014; Soong et al., 2014). Without CD40L data, our understanding of the CD40-CD40L interaction and its effects on immune activation is incomplete. Future studies should measure both CD40 and CD40L to fully elucidate their roles in immune modulation and severe COVID-19 outcomes. Including these measurements will provide deeper insights into the pathophysiology of the disease and inform better therapeutic strategies. Furthermore, a recent study investigating immune checkpoint molecules and the expression of CD39 demonstrated that the co-expression of TIM-3 and CD39 was a significant predictor of severe COVID-19 (Gambichler et al., 2024). These cellular expressions could serve as important prognostic markers.

Additionally, TIM-3 and LAG-3 are indicators of CD8+ T cell exhaustion and are associated with the overexpression of immune inhibitory factors due to T cell exhaustion during SARS-CoV-2 infection (Wherry and Kurachi, 2015; Diao et al., 2020). Through this study, which expanded the number of patients infected with SARS-CoV-2, we were able to confirm that sICs, such as CD27, CD40, and TIM-3, have survival prediction significance. These results are expected to provide supporting evidence for the potential use of sICs as diagnostic and prognostic factors for viral infections, including those caused by SARS-CoV-2. One important consideration is that the sample collection period for this study coincided with the subsequently emerging omicron variant, which has higher transmissibility than the delta variant, is associated with lower clinical severity (Shrestha et al., 2022; Wolf et al., 2023). SARS-CoV-2 infection evolves depending on the timing of infection, exhibiting different host innate immune responses for each variant with distinct characteristics (Wolf et al., 2023). The values of sICs and CKs analyzed in this study may also show differences in quantification compared to those of patients collected during different infection periods. Therefore, further research is required to longitudinally compare the sICs and CKs values for each time period. Furthermore, the 14 and 50 patients included in this paper have cancer and hypertension respectively, as an underlying disease. We compared the sICs values based on cancer and/or hypertension status to exclude the impact of their sICs values on the analysis of COVID-19. Statistical analysis using the Mann-Whitney test showed no significant differences in any of the sICs. Although the clinical utility of sICs is currently limited due to constraints such as testing costs and turnaround time, it is anticipated that the use of sICs in clinical practice for viral infections as disease progression and/or prediction markers will expand in the future.

In this study, ML was used to more clearly and comprehensively investigate the clinical significance and prognostic impact of these markers. To the best of our knowledge, only a few studies have discriminated the significance of sICs and CKs using ML. Various artificial intelligence algorithms, including ML, are expected to be used in various ways as decision-making tools in the medical field (Wang K. et al., 2021; Wang L. et al., 2021; Feng et al., 2022; Hu et al., 2022; Shehab et al., 2022; Sorayaie Azar et al., 2022). In this study, we utilized K-means clustering to handle high-dimensional data, simplifying the analysis and further stratifying patient cohorts. This approach indirectly enables the identification of the most informative markers, as the centroids of the clusters represent the average characteristics of the data points within each cluster (Shehab et al., 2022). Moreover, the ML algorithms DT and SHAP were used as auxiliary prediction tools for patients diagnosed with SARS-CoV-2 infection. DT is a supervised learning method that can predict and classify large datasets using simple logic (Lundberg et al., 2020; Sorayaie Azar et al., 2022; International BR, 2023). SHAP also provides consistent interpretability, helps elucidate the decision-making process, and determines the significance of each feature in the prediction (The Lancet Respiratory M, 2018; Watson et al., 2019). ML analysis has the advantage of not only accurately classifying and predicting specimens through learning from observations and logic, but also enabling decision-making, excluding subjective factors. In this study, IL-6 showed the highest importance in predicting mortality using both the DT and SHAP methods. This aligns with the outcomes of the ROC curve analysis, which showed that IL-6 had the highest AUC value among all factors, including existing infection markers such as CRP and PCT. It has been reported that IL-6 induces disease, is related to CK storm, and is helpful in predicting survival prognosis (Copaescu et al., 2020; Gubernatorova et al., 2020; Majidpoor and Mortezaee, 2022). The results of this study, which included a larger number of individuals with SARS-CoV-2 infection than previous studies and utilized both ML and performance analyses, further support the value of IL-6 in clinical practice. Additionally, through DT and SHAP, it was observed that sCD27, sCD40, and sTIM-3 among sICs and IL-13 and TNF-alpha among CKs were highly important in survival prediction. In the future, for the prediction of mortality from SARS-CoV-2 infection, the use of sICs and CKs, in addition to existing infection markers, could be helpful. IL-6 is expected to be the most effective marker for survival prediction.

In addition, in this study, it was possible to perform a paired t-test analysis on the difference in values between the 1^st^ and 2^nd^ weeks in the expanded patient group and discriminate sICs and/or CKs that significantly increased or decreased in the second week. sICs, such as sCD27, sCD40, aTIM-3, and sLAG-3, and CKs, such as IL-13, which were found to have a high correlation with prognosis in this study, showed significant differences over time. In contrast, IL-6 levels did not show a significant difference between the first and second weeks, and increased to high levels even in the early stages of infection. IL-6 has been previously reported as an early increase marker (Waje-Andreassen et al., 2005), and is widely recognized for its pivotal role in severe inflammatory responses, including the cytokine storm (Grebenciucova and VanHaerents, 2023). Additionally, sCD27 and sCD40 have been shown to play significant roles in immune responses in conditions such as sepsis and viral infections (Lee et al., 2022), while sTIM-3 has been linked to T cell exhaustion, particularly in chronic viral infections and cancers (Paranga et al., 2024). However, despite the substantial research on their clinical relevance, there are limited reports on the temporal dynamics of IL-6 elevation during SARS-CoV-2 infection. The results of this study suggest that IL-6 is a valuable prognostic marker that increases significantly even in the early stages of infection.

The limitations of this study were as follows. First, it was affected by a selection bias owing to its retrospective nature. If the predictive model is validated in a multicenter cohort in future follow-up studies, the predictive ability of the ML algorithm is expected to become more reliable. Second, as a comparison with healthy controls was not possible for the measurement of sICs and CKs, a more objective evaluation would be possible if additional comparisons with healthy controls were conducted in future research. In addition, most non-survivors in this study (16 of 19) were classified as pneumonia-related deaths and the analysis of the prognosis among patients with pneumonia was limited compared to that of deceased patients. Furthermore, the overall number of non-survivors is relatively low compared to the number of survivors. This disparity may be partially attributed to the clinical manifestations associated with the Omicron variant. It is necessary to conduct follow-up studies involving more non-survivor patients. Third, we aimed to elucidate the relationship between co-infection with viruses, bacteria, and fungi in COVID-19 and sIC. However, it was challenging to determine statistical significance because of the limited number of patients infected with multidrug-resistant bacteria. We intend to continue this research in follow-up studies.

In conclusion, this study simultaneously measured various sIC and CK levels along with previous infection markers in hospitalized patients with SARS-CoV-2 infection and designed a process that could be used in actual clinical practice by utilizing the ML algorithm. sICs and CKs are expected to be more actively used in the future for the prognostic prediction of viral infections, including SARS-CoV-2. This study is expected to be helpful as a basis for the appropriate selection of factors for use as markers in medical practice among the numerous types of sICs and CKs in patients with severe form of SARS-CoV-2. Further research is essential, particularly increasing the sample size to enhance the study’s robustness and statistical power. Larger cohorts will improve reliability and allow precise evaluation of the clinical utility of various sICs and CKs, advancing prognostic predictions for viral infections in clinical practice.

## Data Availability

The datasets presented in this study can be found in online repositories. The names of the repository/repositories and accession number(s) can be found below: Harvard Dataverse https://doi.org/10.7910/DVN/12D49O.
