# Supplementary material for: Predicting survival in patients with SARS-CoV-2 based on cytokines and soluble immune checkpoint regulators
Source: Front Cell Infect Microbiol. 2024 Nov 25;14:1397297. doi: 10.3389/fcimb.2024.1397297 (PMC11625743; doi:10.3389/fcimb.2024.1397297)
Supplement: Supplementary file 1 [file Table1.docx]

Supplementary Material

## Supplementary Figures

## Supplementary FIGURE 1| Kaplan–Meier curves of soluble immune checkpoints (sICs) and cytokines (CKs) for the overall survival in patients infected with SARS-CoV-2.

**Supplementary FIGURE 2**| K-means clustering analysis for combination of soluble immune checkpoints (sICs), cytokines (CKs), inflammatory markers including C-reactive protein, procalcitonin, and neutrophil-to-lymphocyte ratio.

**Supplementary FIGURE 3**| Comparison of the paired sICs and CK values between 1^st^ and 2^nd^ weeks with SARS-CoV-2 infection.

## Supplementary Table

**Supplementary TABLE 1|** Correlation between inflammatory markers and soluble immune checkpoint regulators (sICs) and cytokines


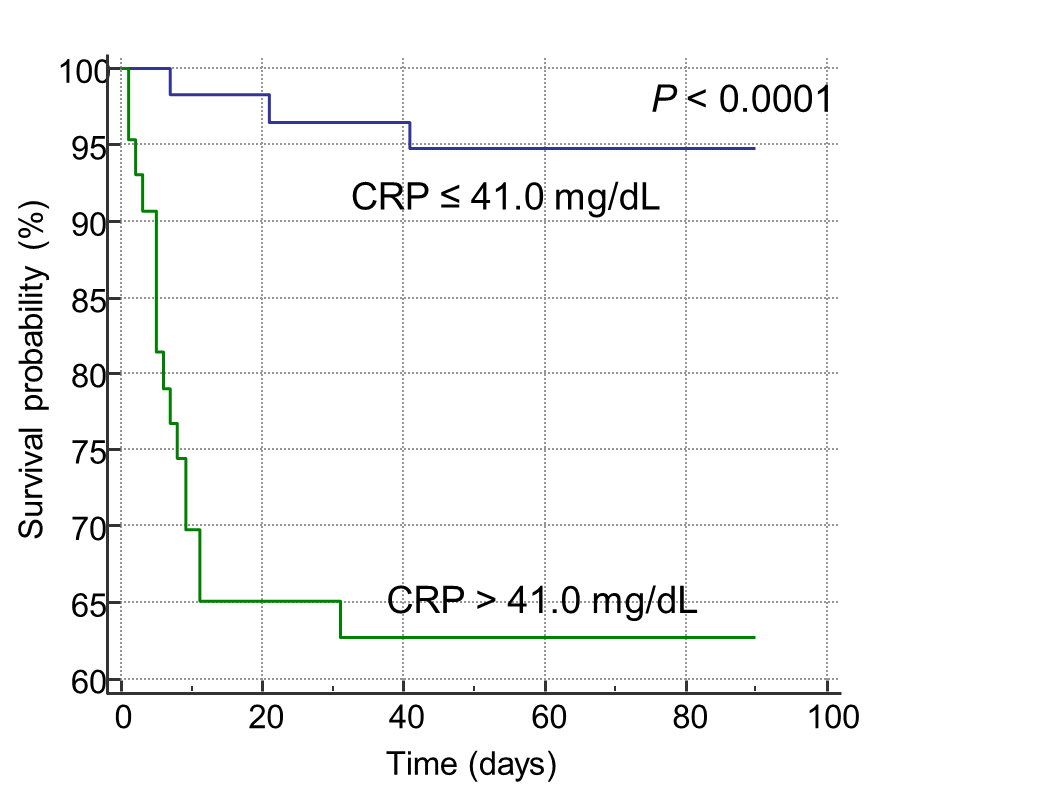

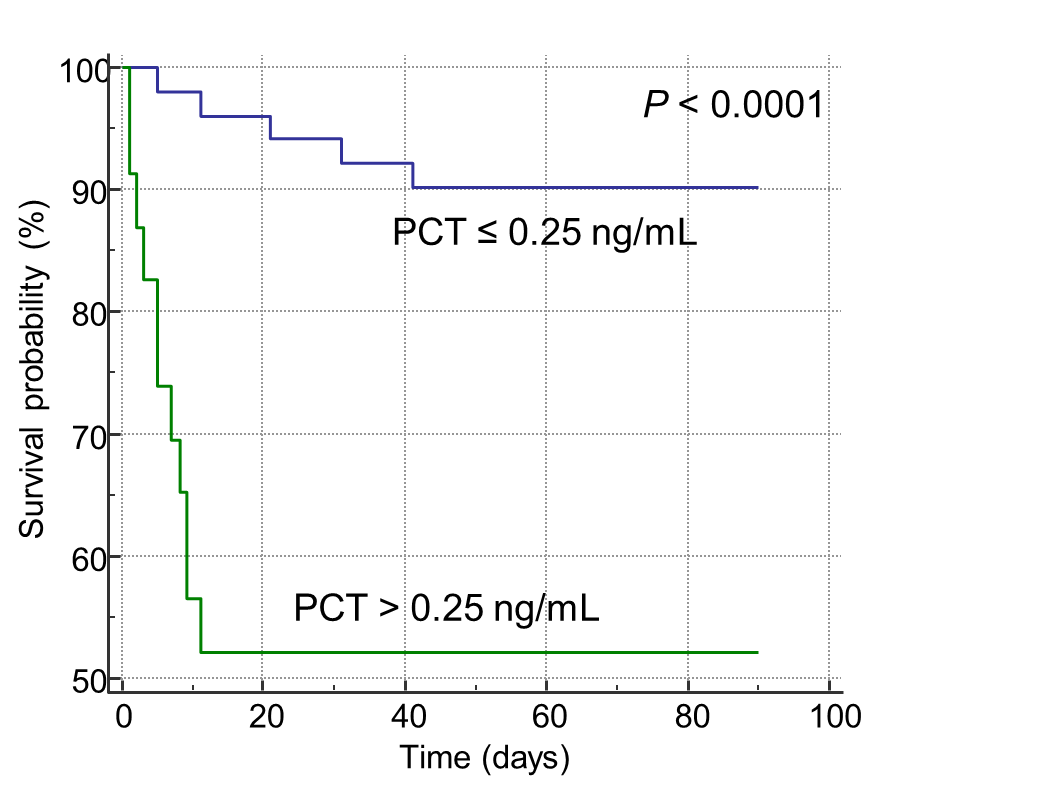

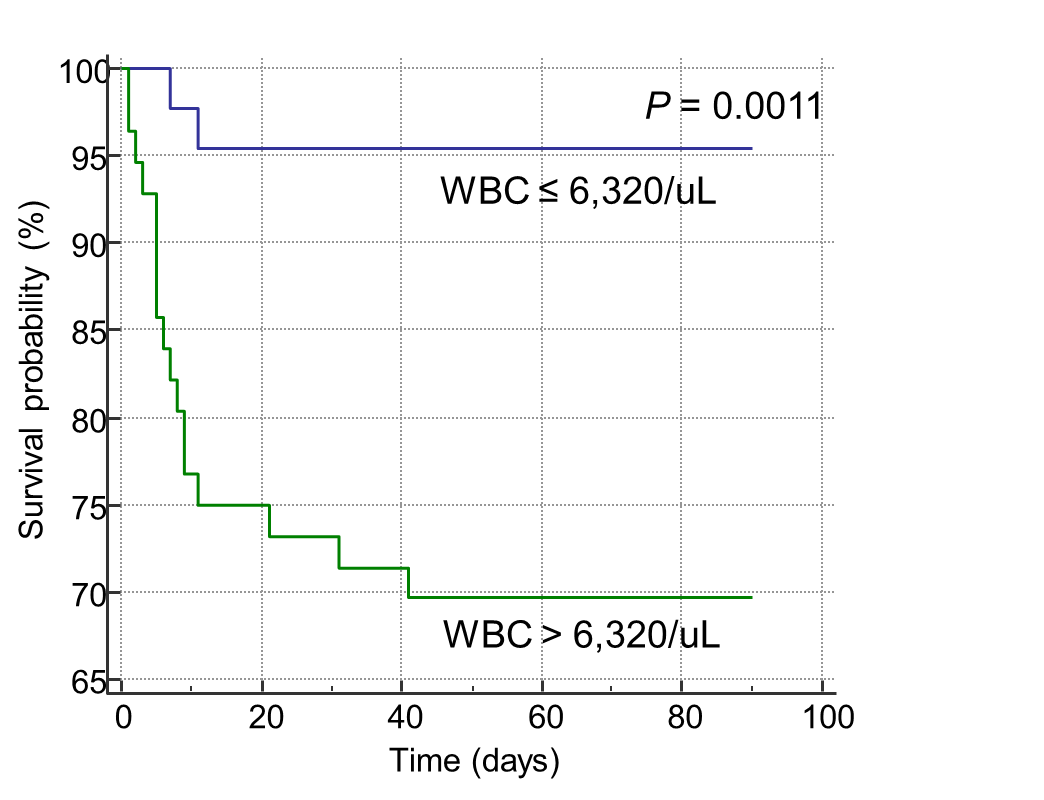

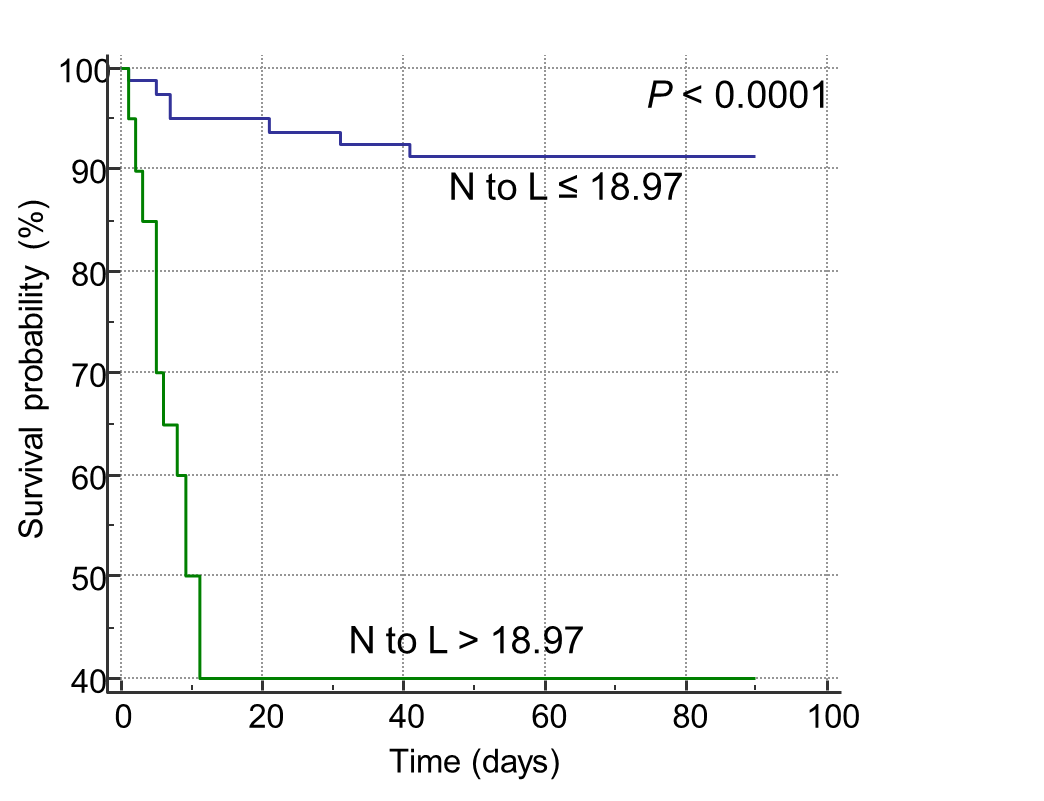


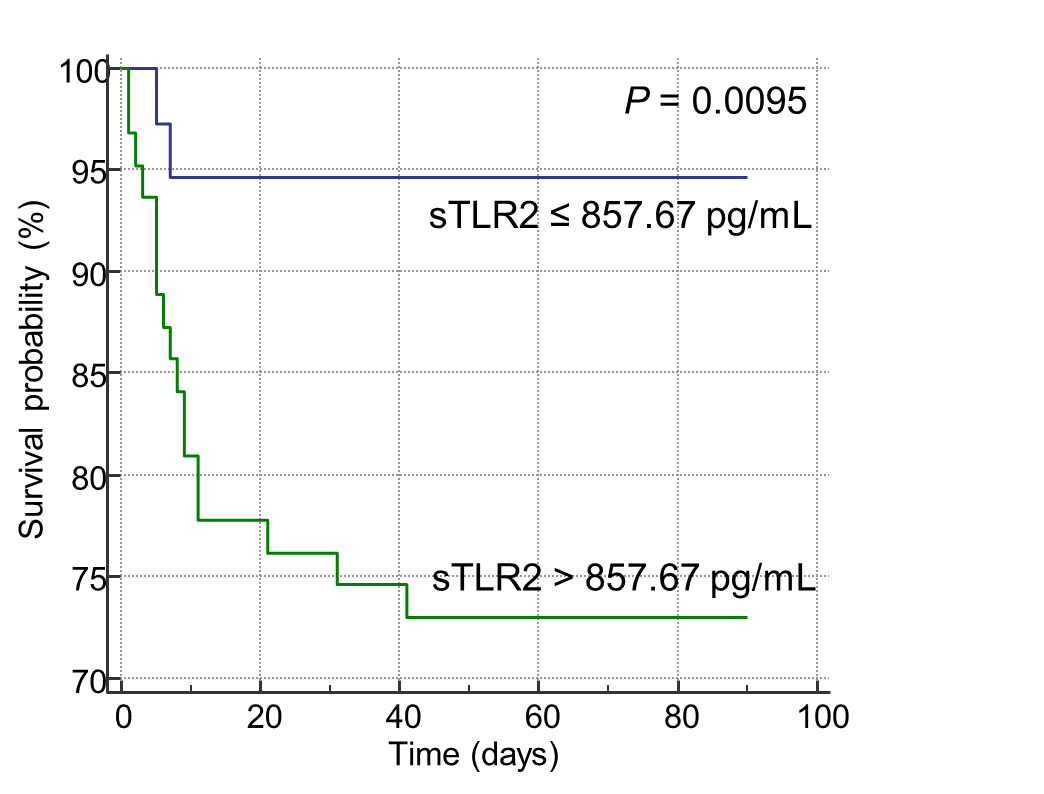

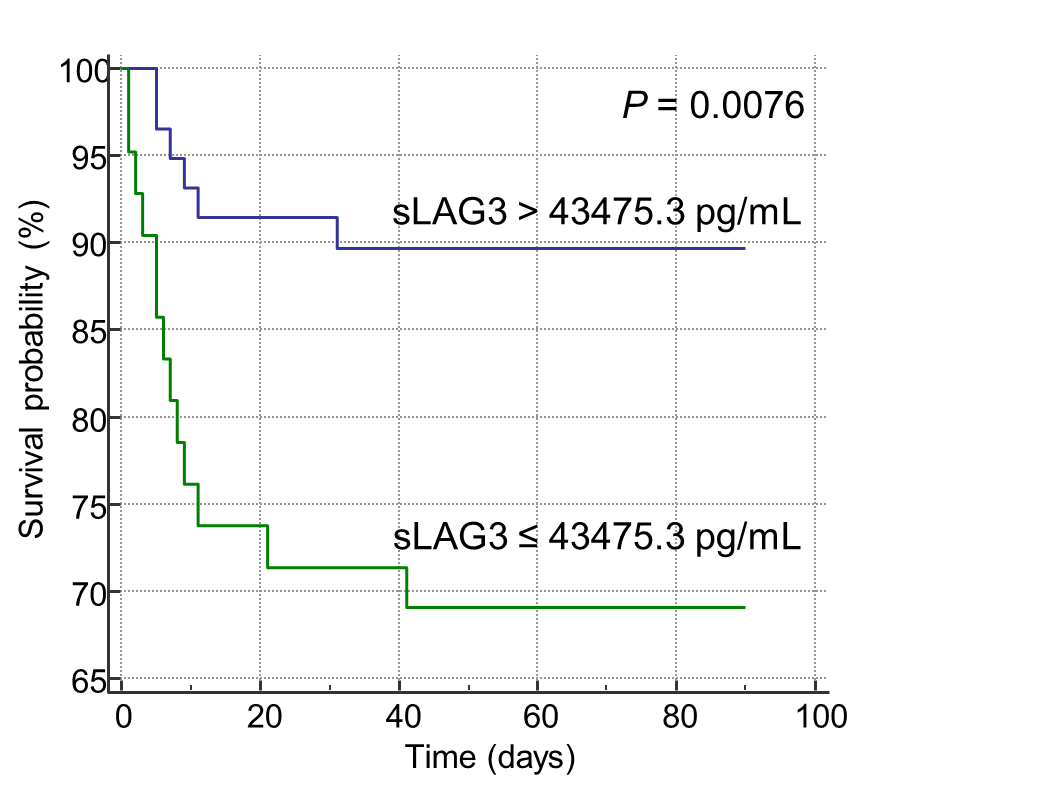


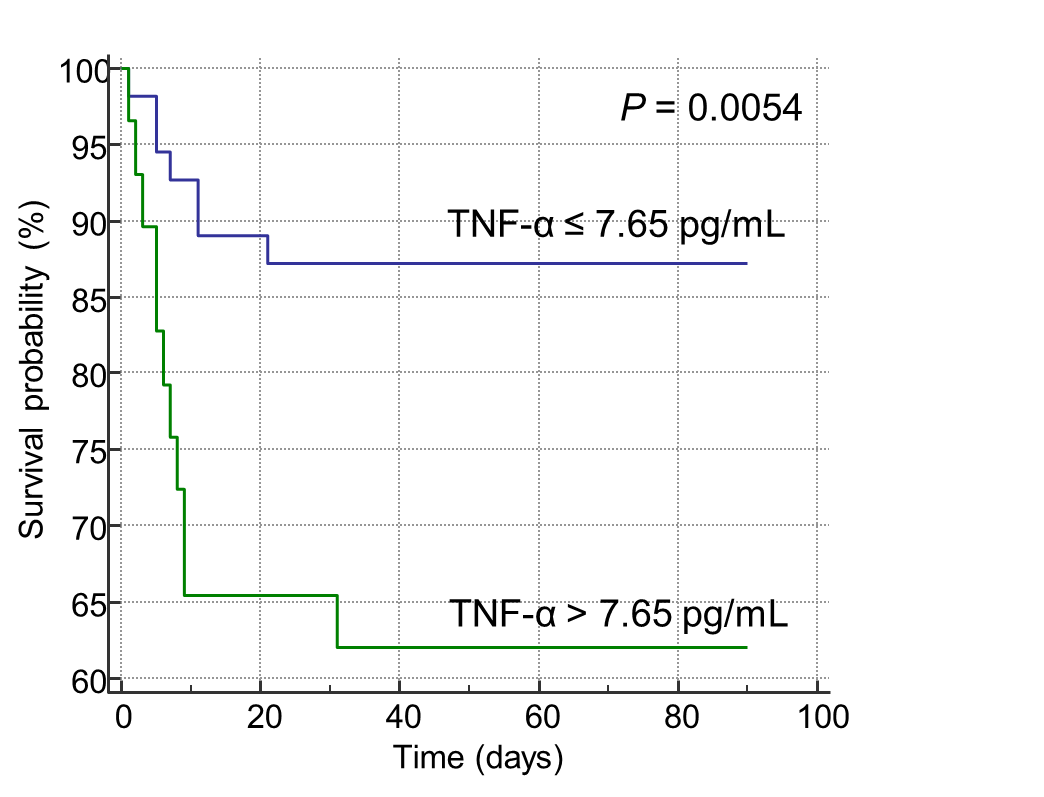

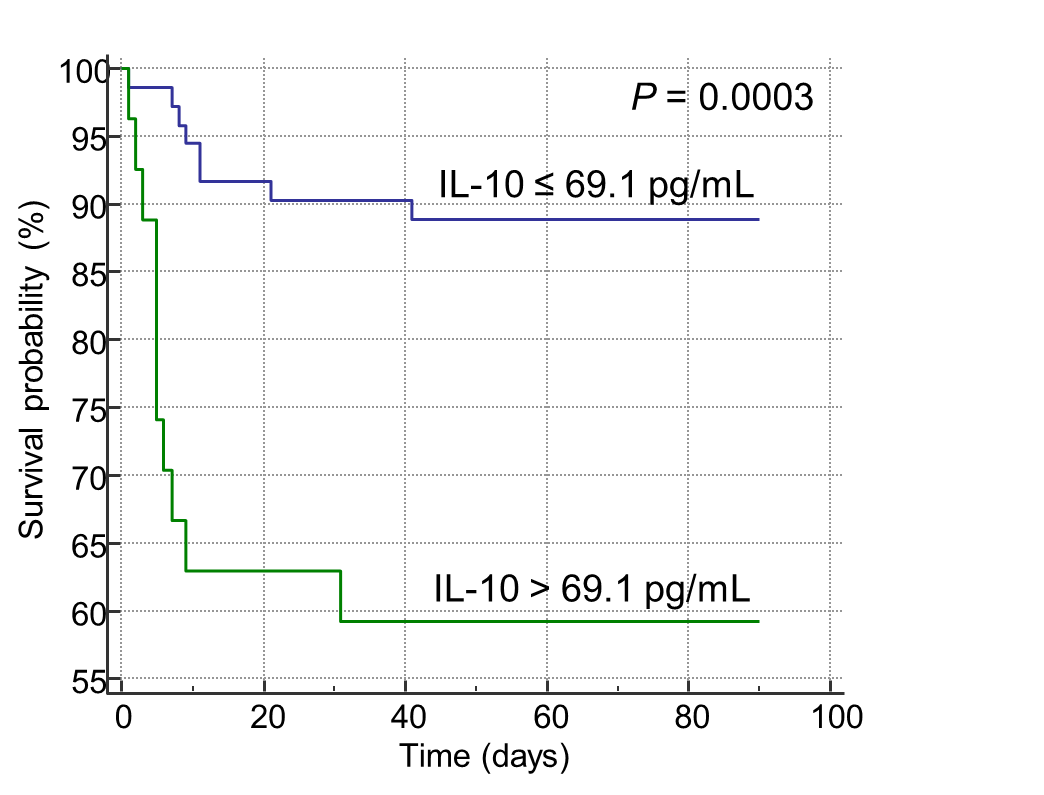

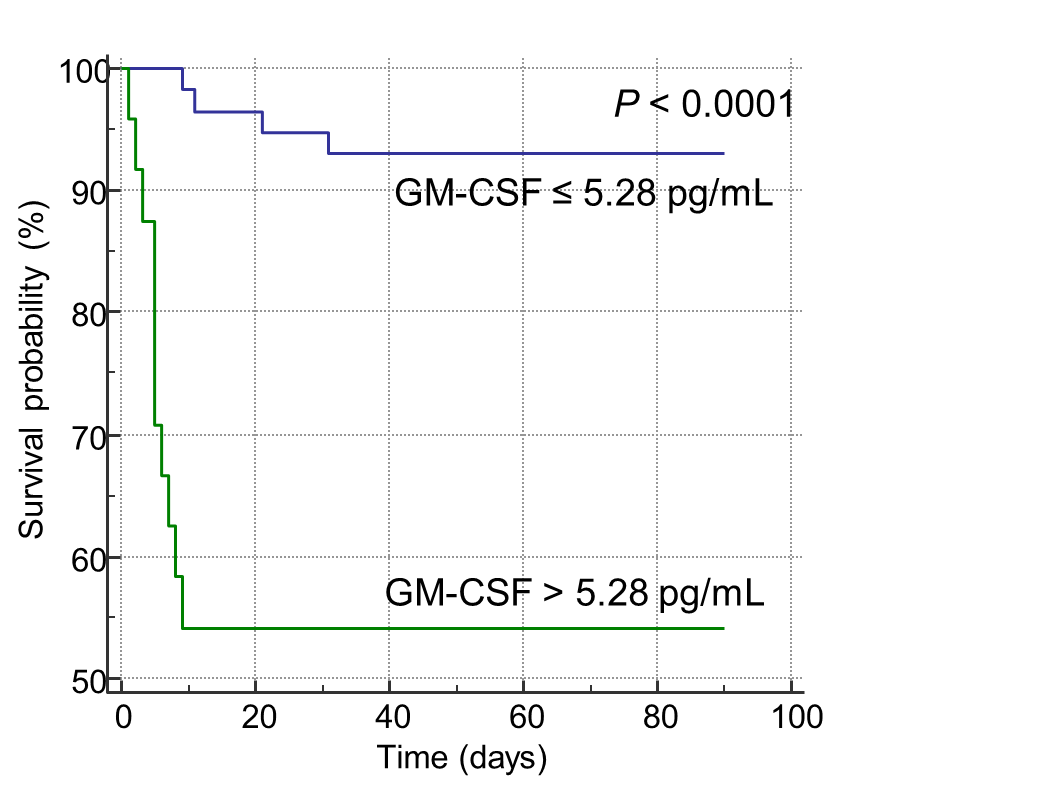

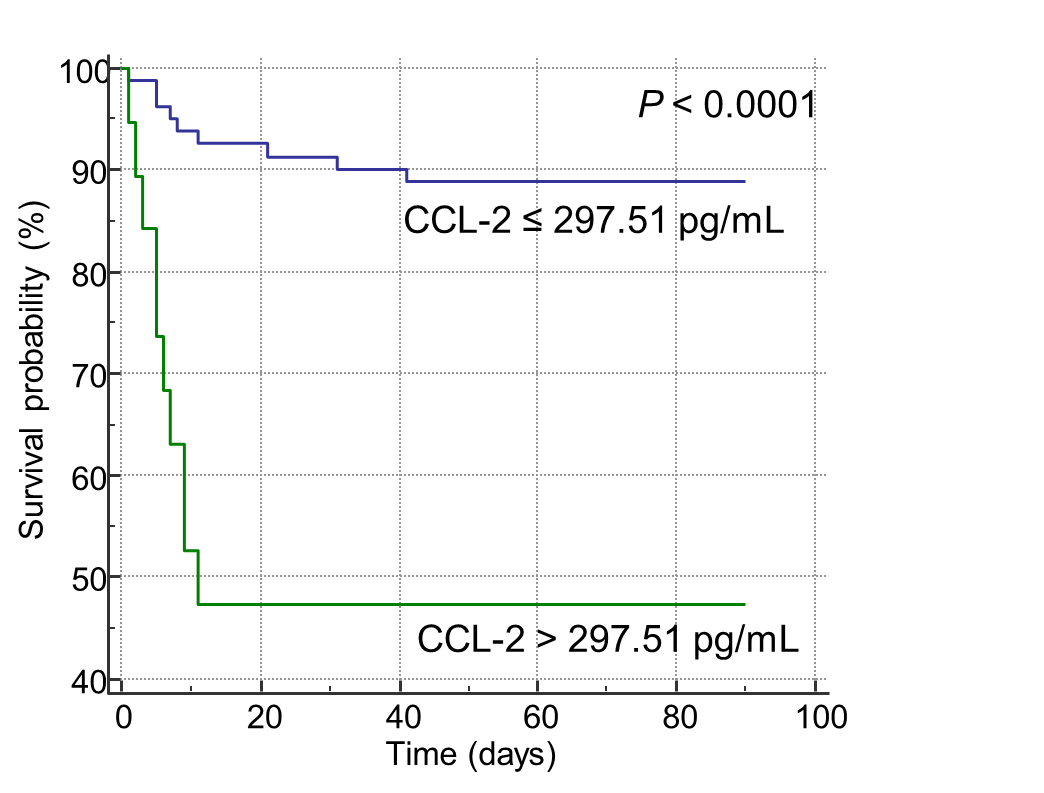


Supplementary FIGURE 1| Kaplan–Meier curves of soluble immune checkpoints (sICs) and cytokines (CKs) for the overall survival in patients infected with SARS-CoV-2.


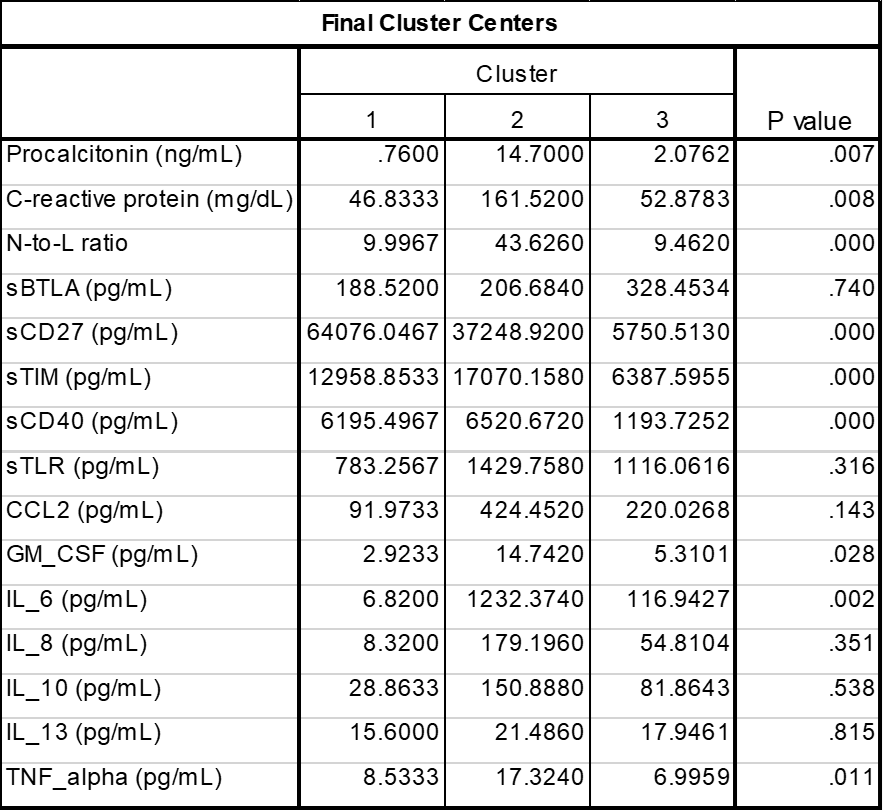

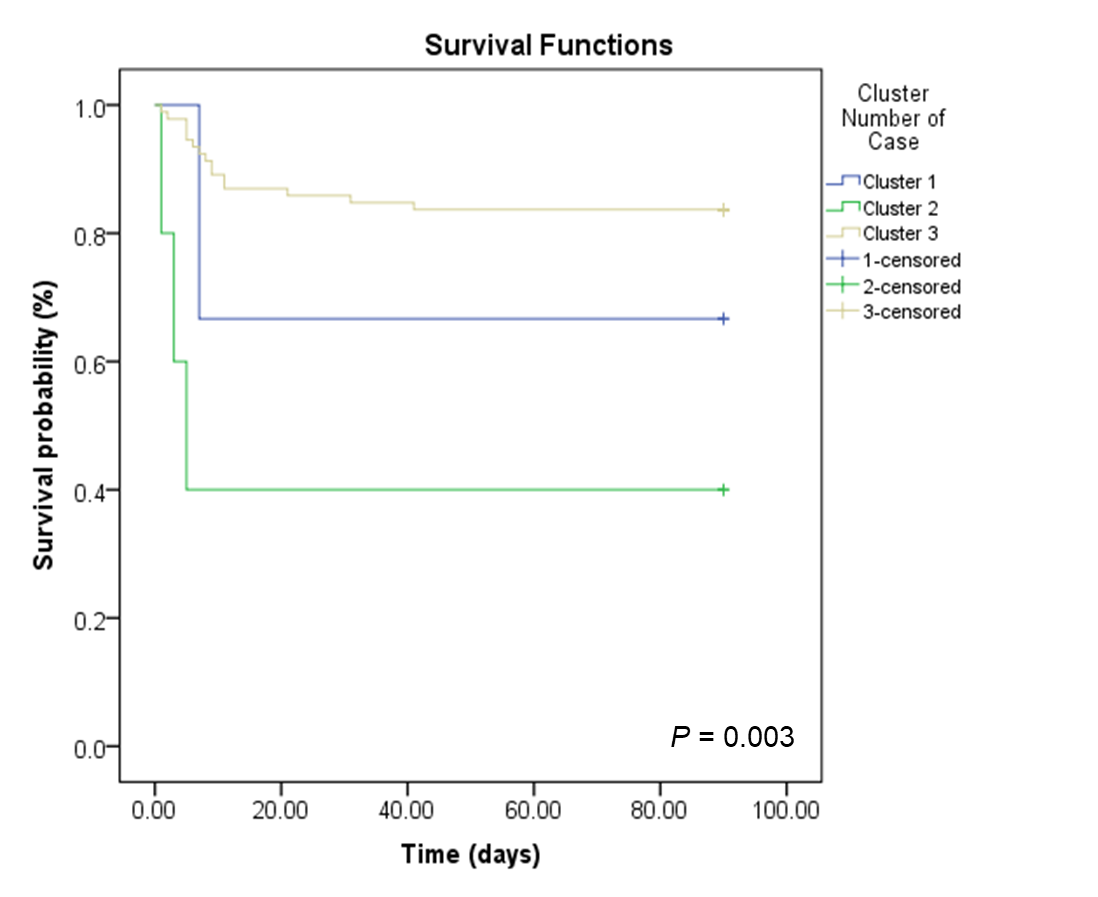


**Supplementary FIGURE 2**| K-means clustering analysis for combination of soluble immune checkpoints (sICs), cytokines (CKs), inflammatory markers including C-reactive protein, procalcitonin, and neutrophil-to-lymphocyte ratio.


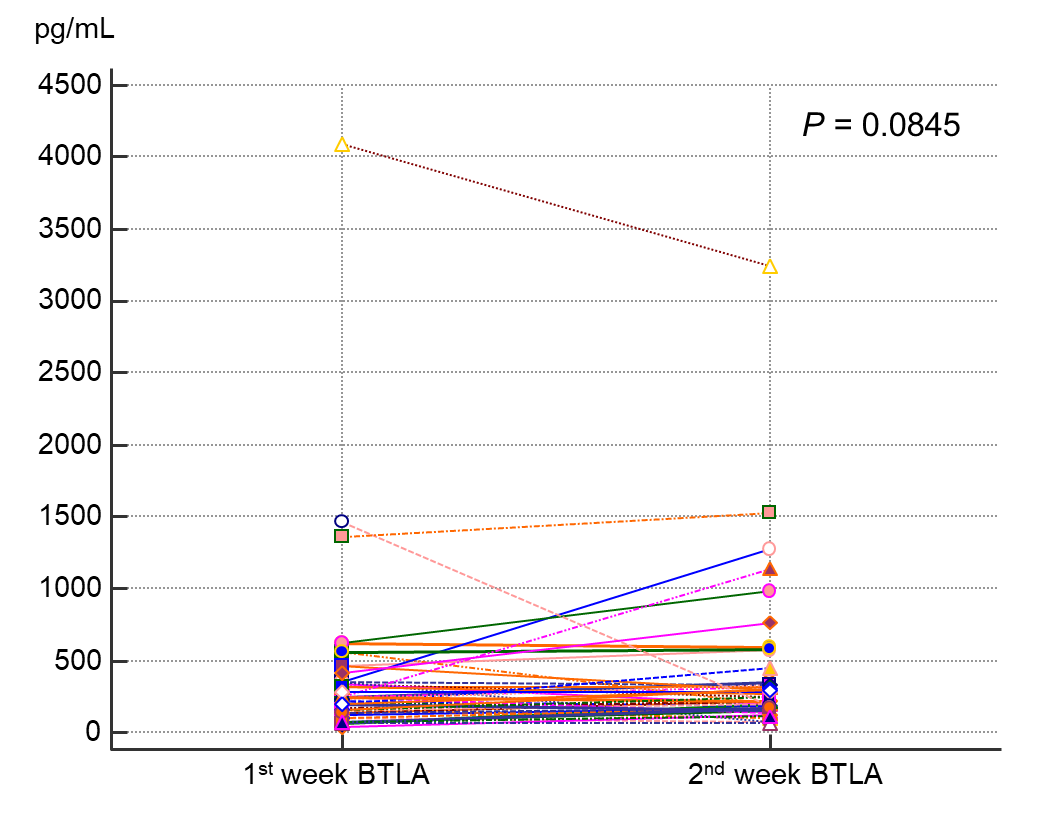


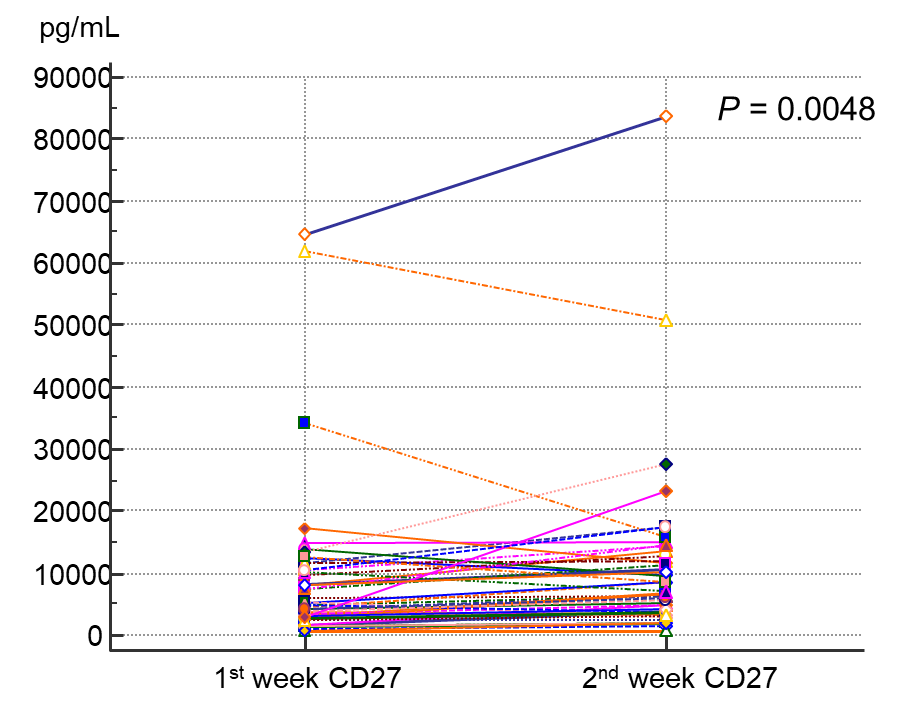


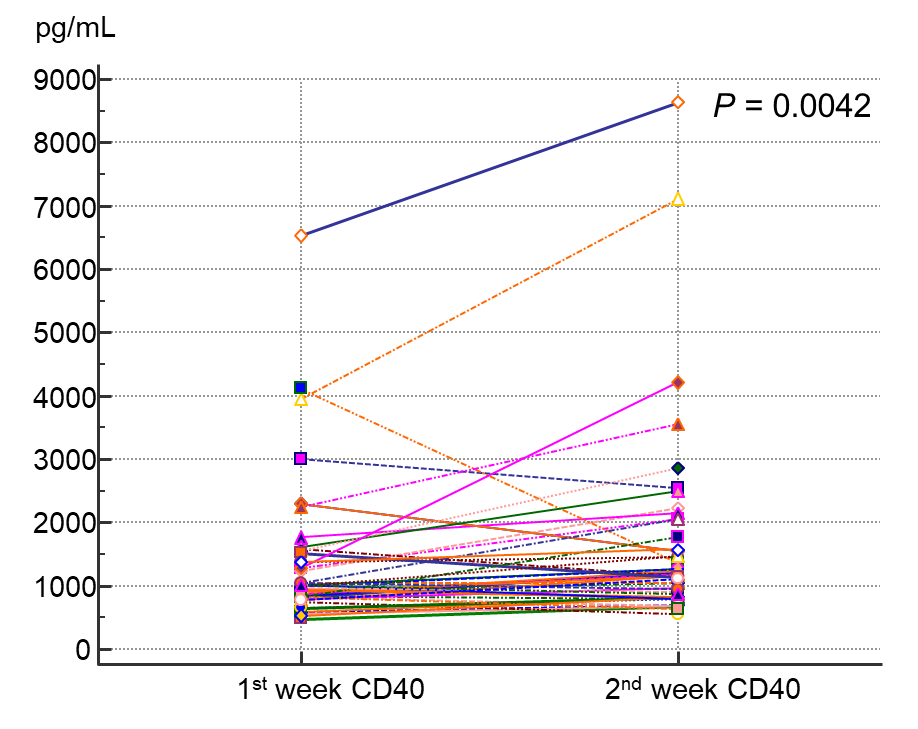

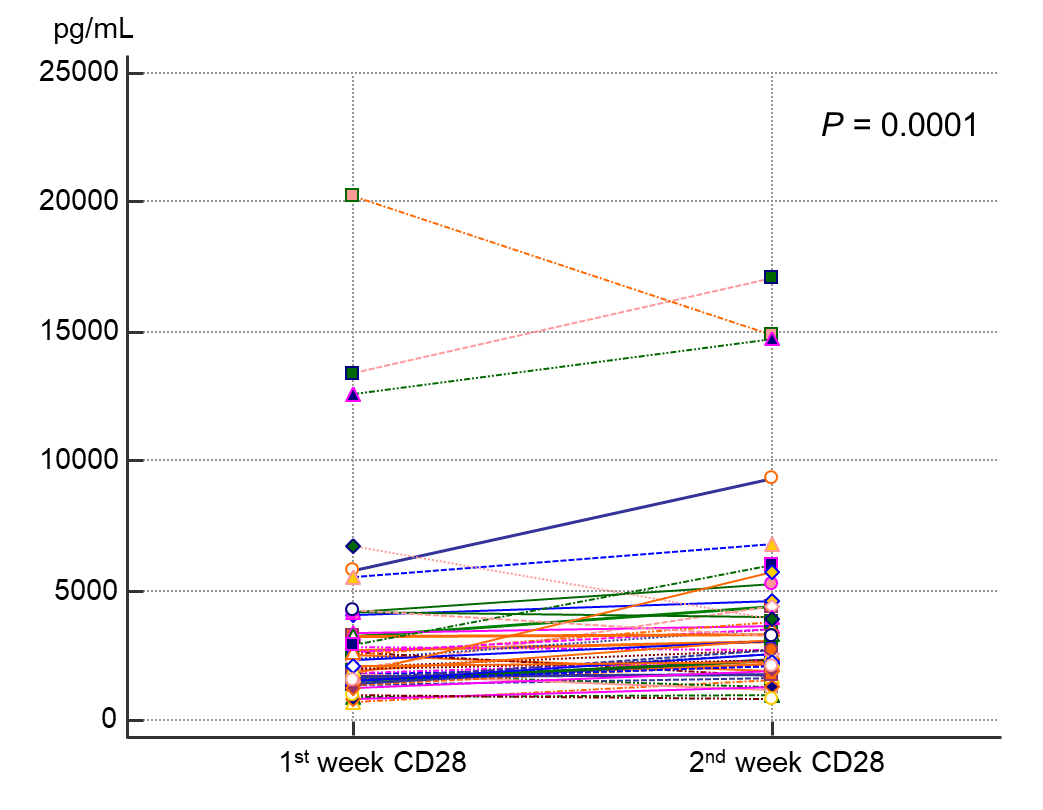


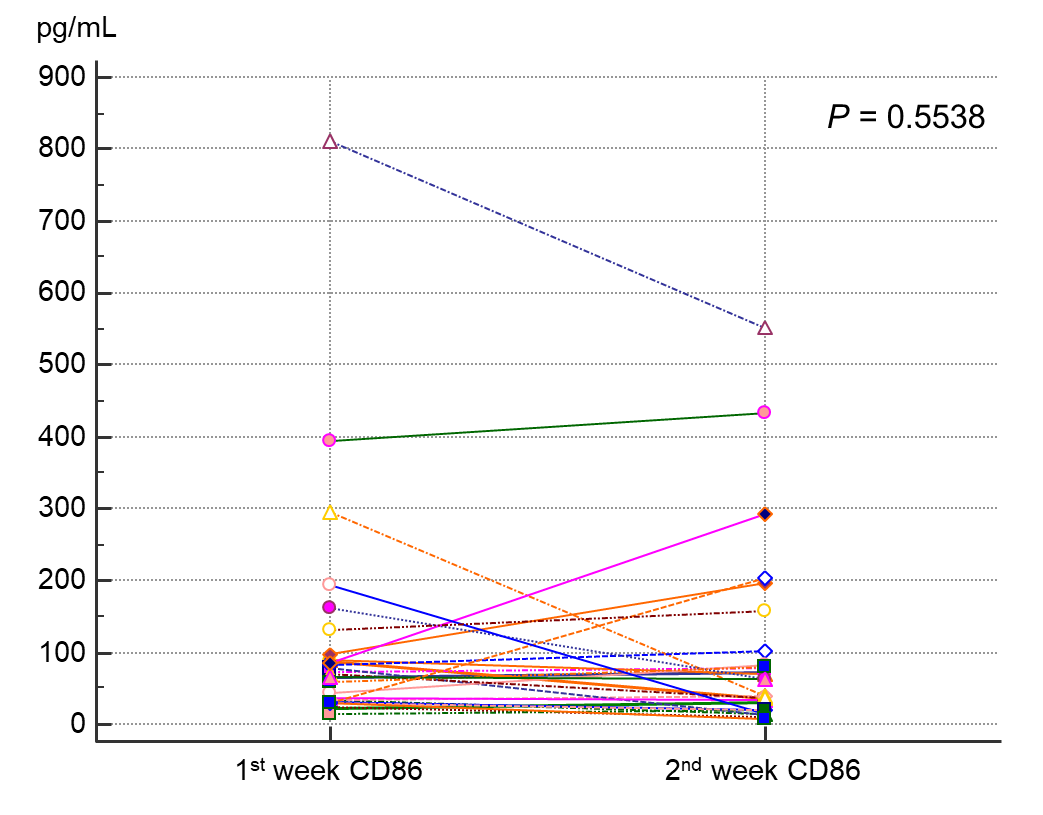

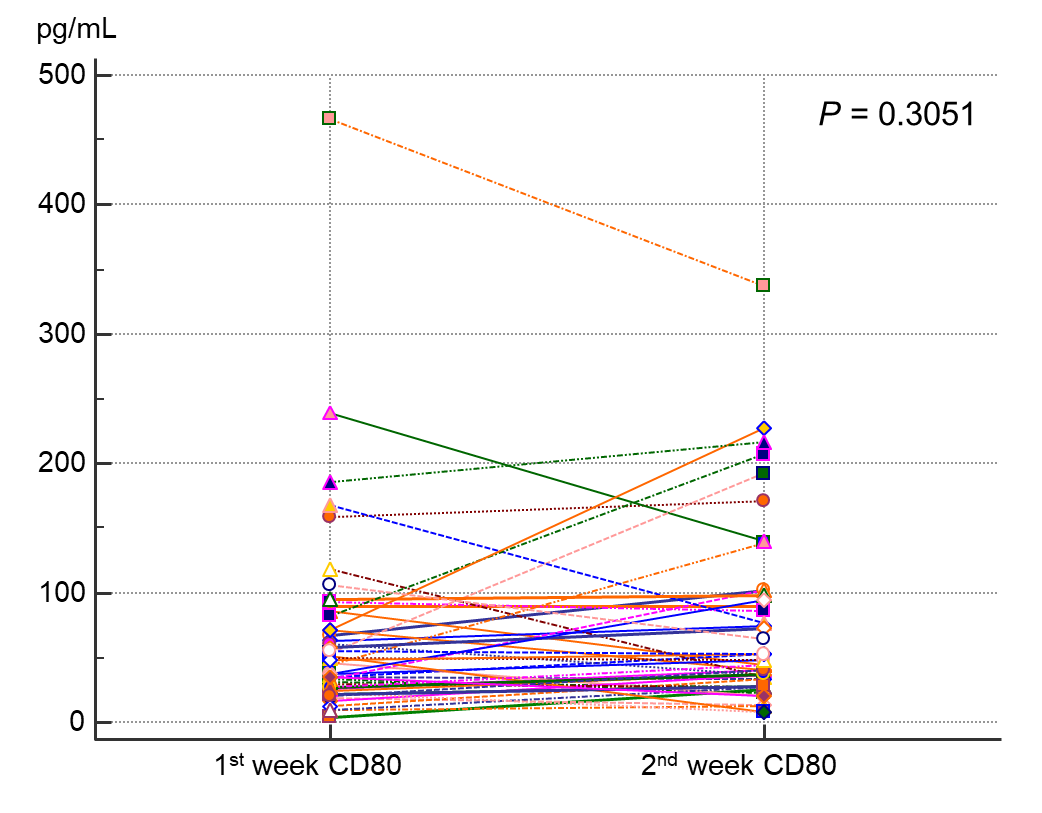


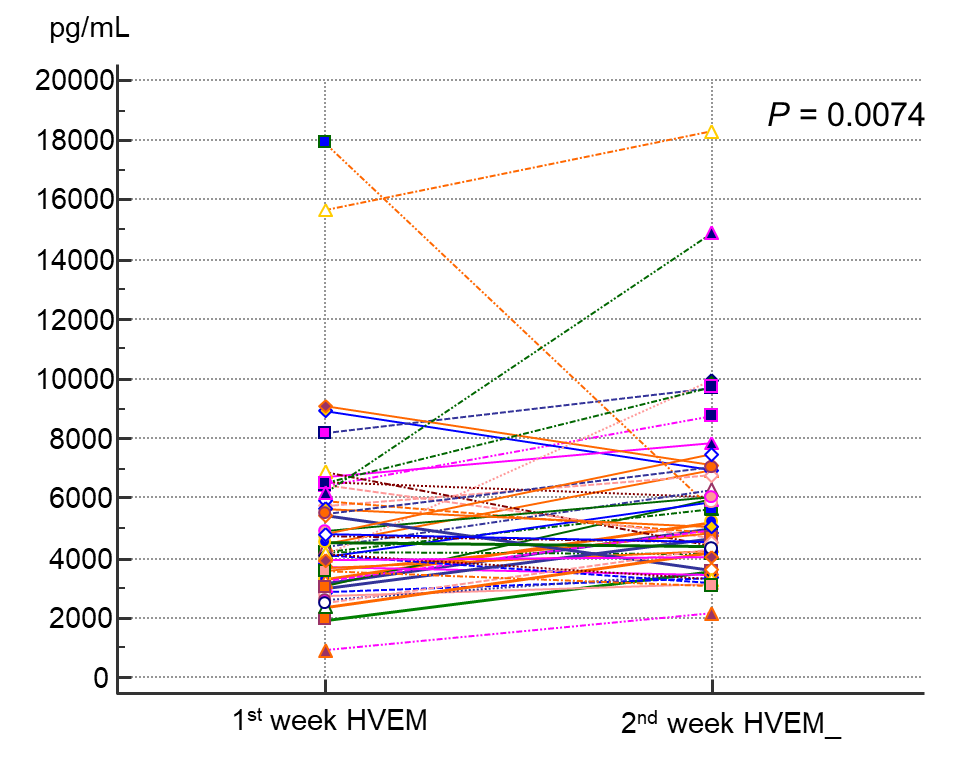

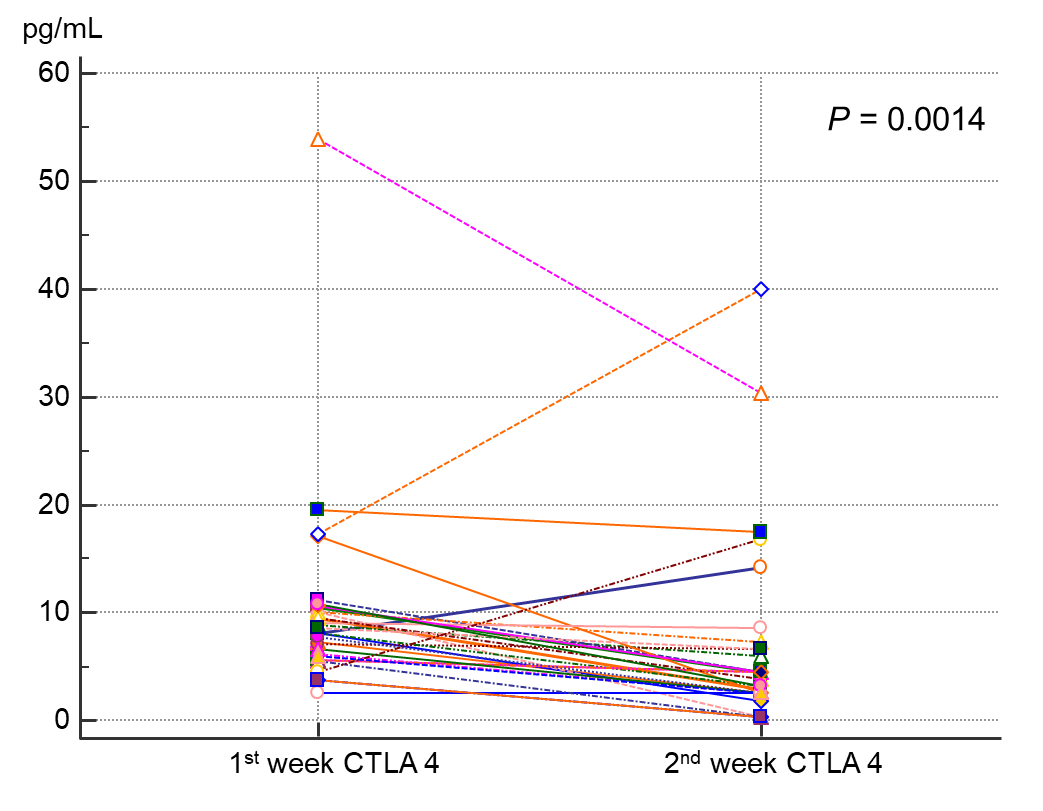


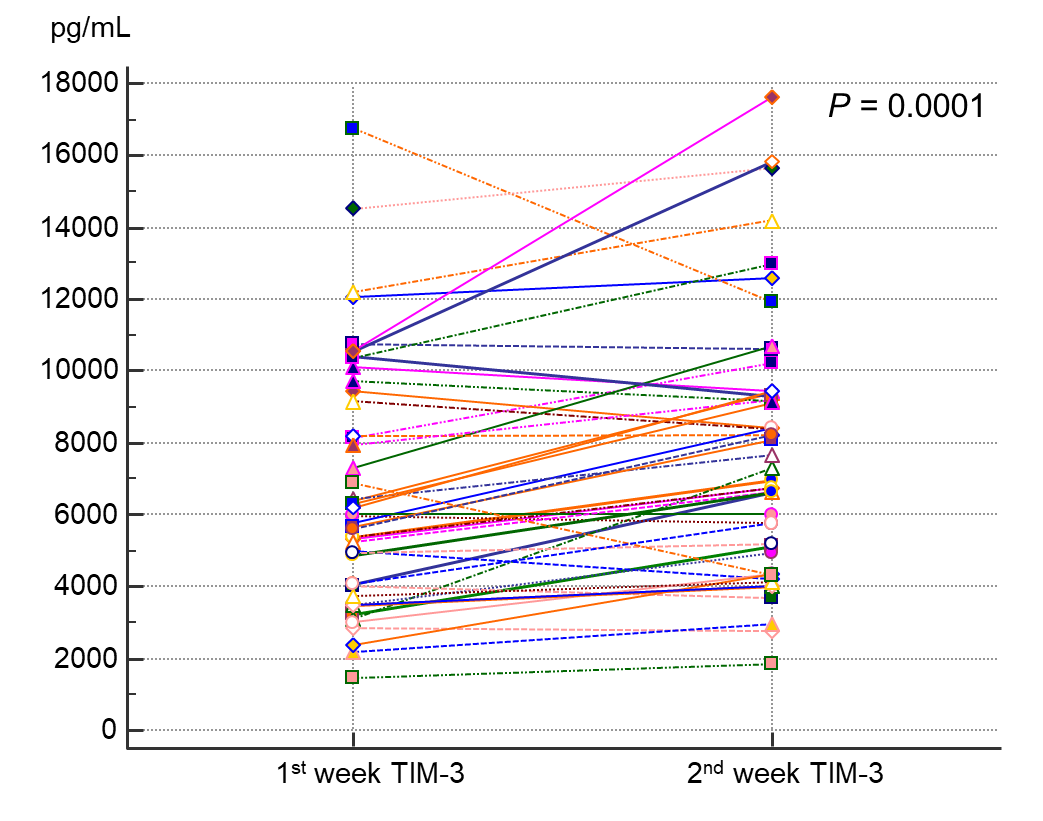

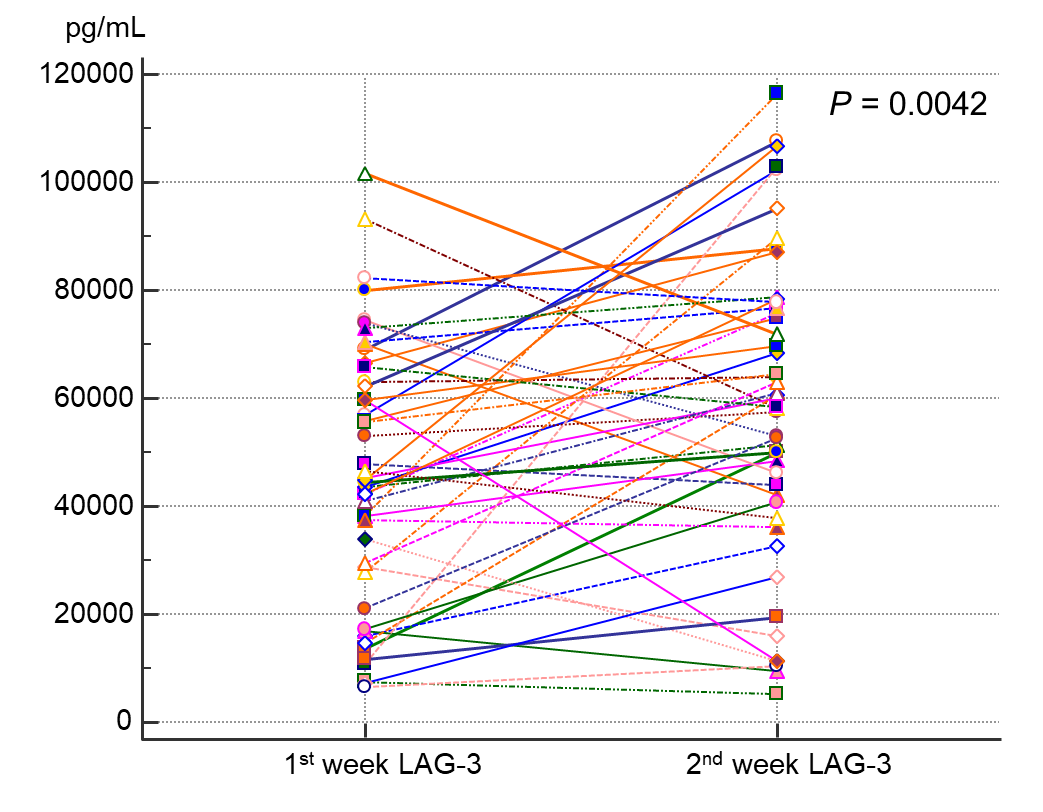


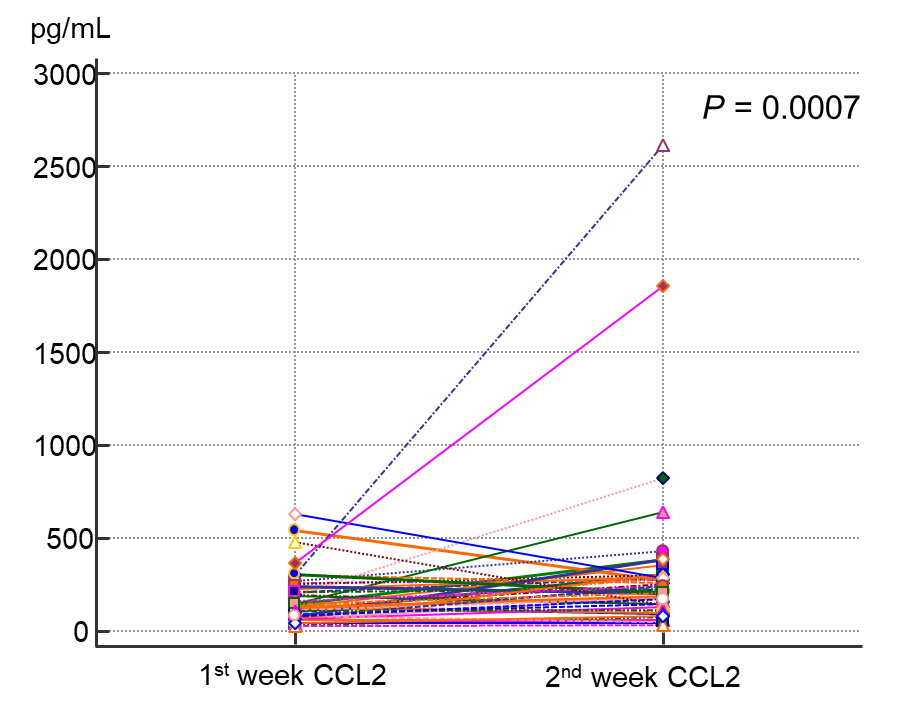

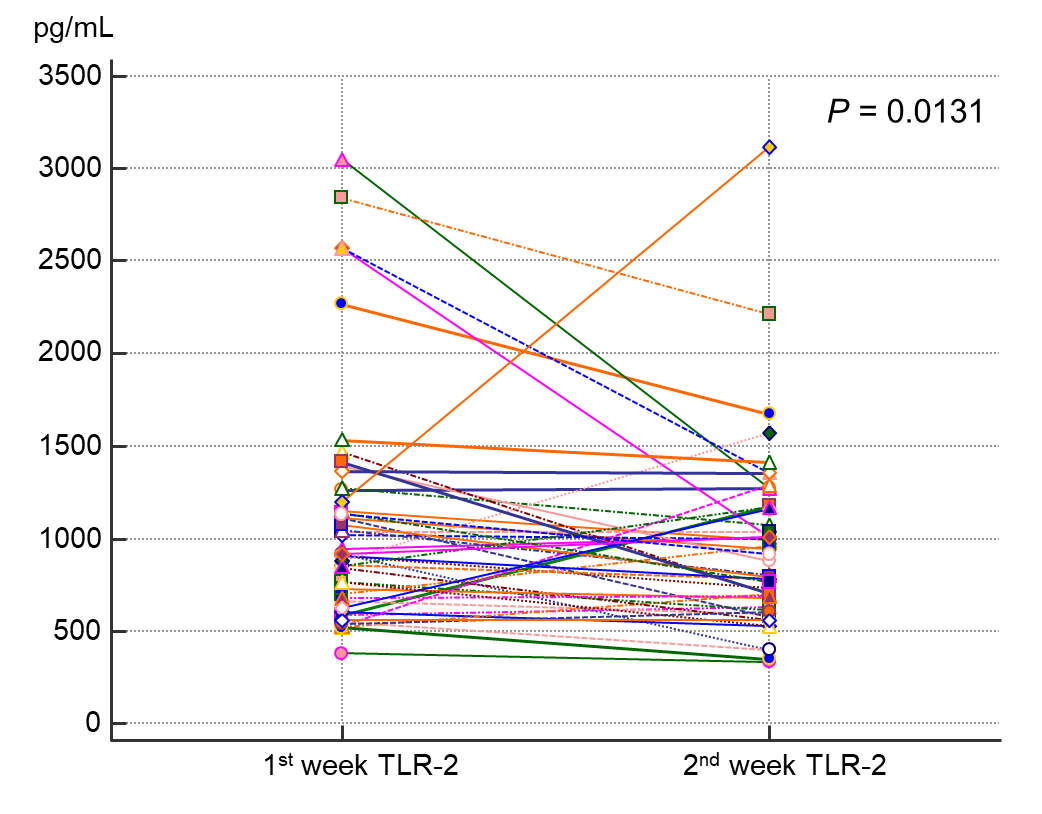


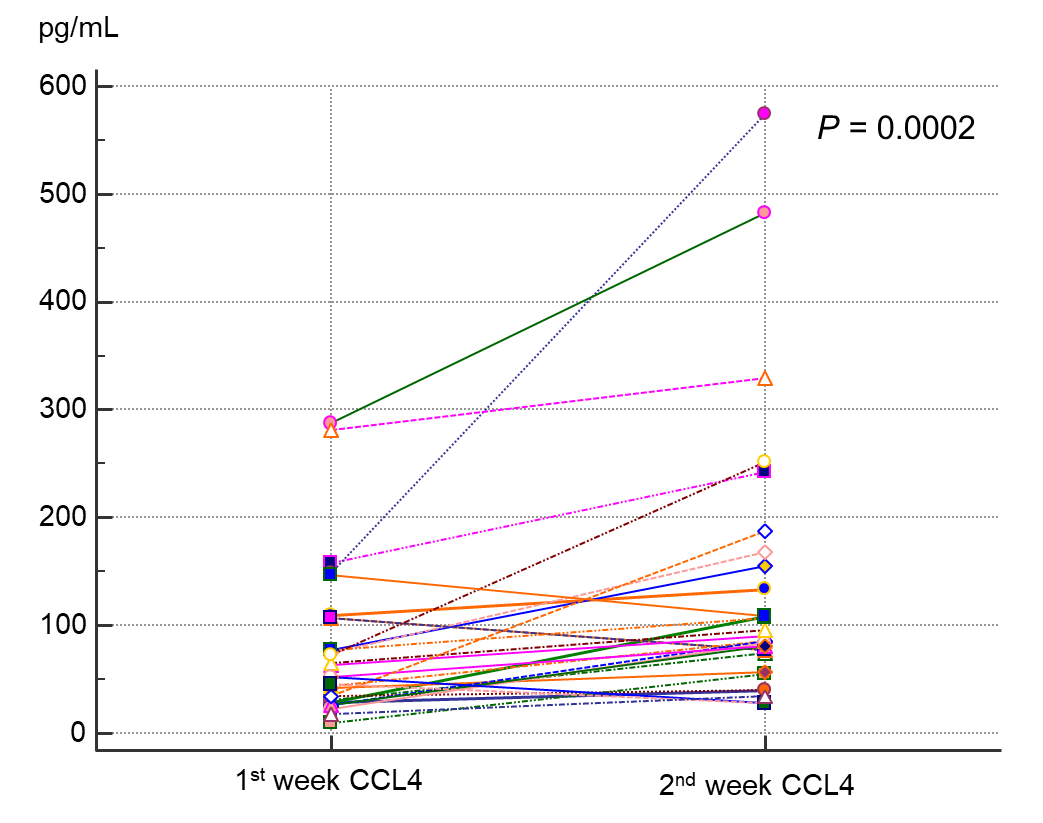

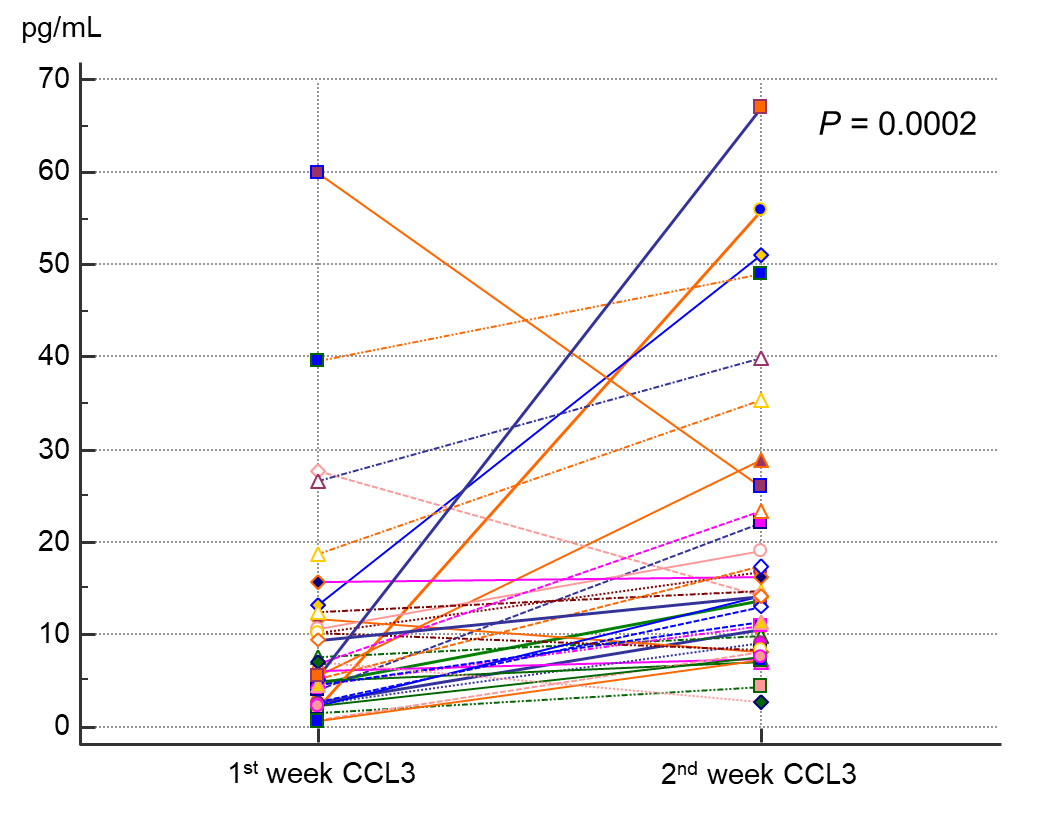


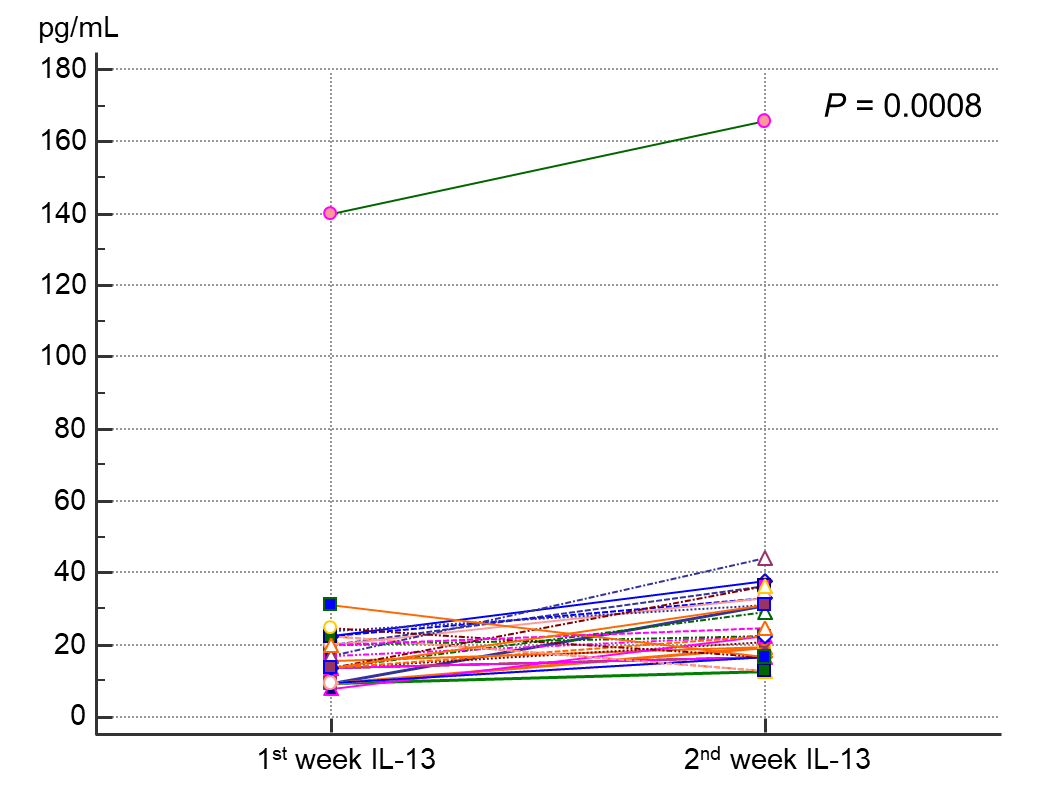

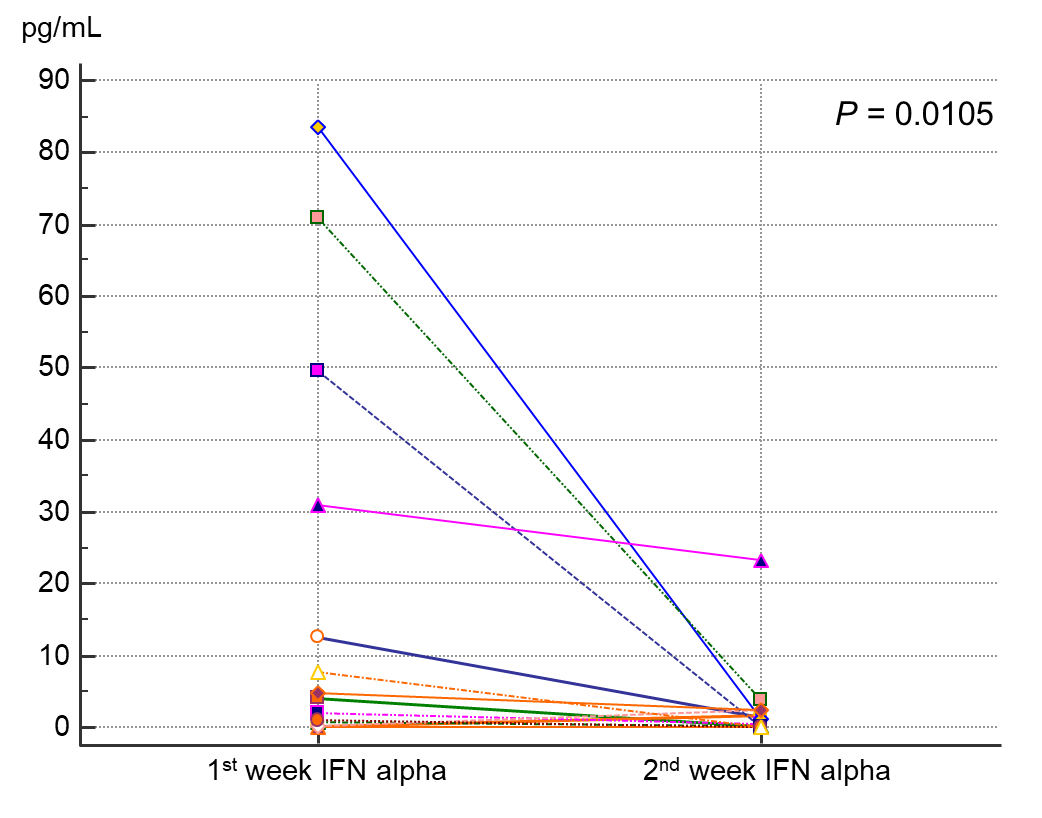


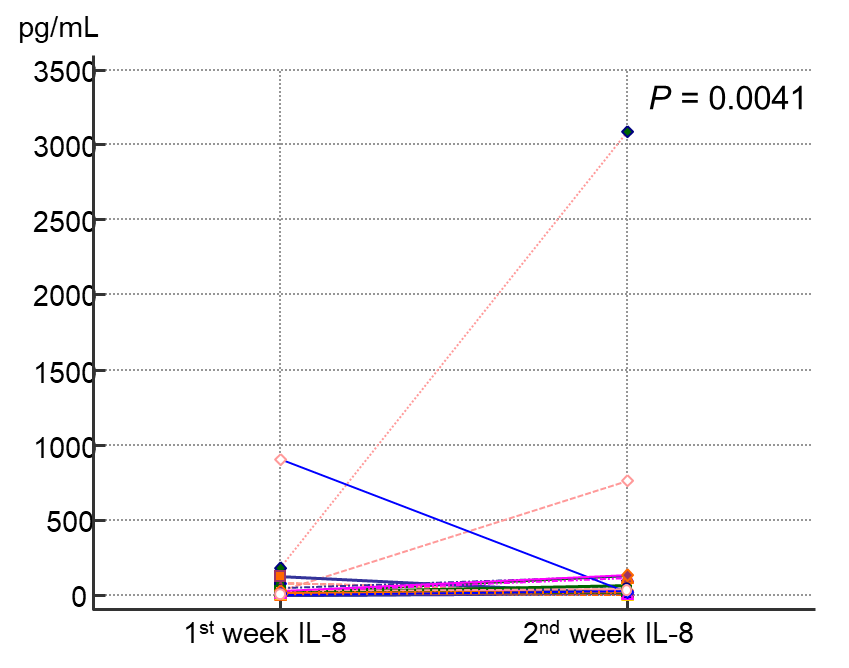


Supplementary FIGURE 3| Comparison of the paired sICs and CK values between 1^st^ and 2^nd^ weeks with SARS-CoV-2 infection.

**Supplementary TABLE 1|** Correlation between inflammatory markers and soluble immune checkpoint regulators (sICs) and cytokines

| **Variables** | | | **CRP** | **PCT** |
| --- | --- | --- | --- | --- |
| **sICs** | **sBTLA** | **Correlation coefficient** | -0.125 | -0.145 |
|  |  | **Significance Level P** | 0.2167 | 0.2285 |
|  |  | **n** | 99 | 71 |
|  | **sCD27** | **Correlation coefficient** | 0.171 | 0.125 |
|  |  | **Significance Level P** | 0.0886 | 0.2947 |
|  |  | **n** | 100 | 72 |
|  | **sCD28** | **Correlation coefficient** | 0.004 | -0.116 |
|  |  | **Significance Level P** | 0.9651 | 0.3324 |
|  |  | **n** | 100 | 72 |
|  | **sCD40** | **Correlation coefficient** | 0.414 | 0.37 |
|  |  | **Significance Level P** | <0.0001 | 0.0014 |
|  |  | **n** | 100 | 72 |
|  | **sCD80/B7-1** | **Correlation coefficient** | -0.211 | -0.162 |
|  |  | **Significance Level P** | 0.0351 | 0.1745 |
|  |  | **n** | 100 | 72 |
|  | **sCD86/B7-2** | **Correlation coefficient** | -0.15 | -0.14 |
|  |  | **Significance Level P** | 0.1585 | 0.2695 |
|  |  | **n** | 90 | 64 |
|  | **sCTLA-4** | **Correlation coefficient** | -0.151 | -0.122 |
|  |  | **Significance Level P** | 0.1415 | 0.318 |
|  |  | **n** | 96 | 69 |
|  | **sGITR** | **Correlation coefficient** | -0.157 | -0.184 |
|  |  | **Significance Level P** | 0.1562 | 0.1677 |
|  |  | **n** | 83 | 58 |
|  | **sGITRL** | **Correlation coefficient** | -0.109 | -0.186 |
|  |  | **Significance Level P** | 0.2885 | 0.1235 |
|  |  | **n** | 97 | 70 |
|  | **sHVEM** | **Correlation coefficient** | 0.359 | 0.318 |
|  |  | **Significance Level P** | 0.0002 | 0.0064 |
|  |  | **n** | 100 | 72 |
|  | **sICOS** | **Correlation coefficient** | -0.167 | -0.088 |
|  |  | **Significance Level P** | 0.0967 | 0.4601 |
|  |  | **n** | 100 | 72 |
|  | **sLAG-3** | **Correlation coefficient** | -0.367 | -0.256 |
|  |  | **Significance Level P** | 0.0002 | 0.0298 |
|  |  | **n** | 100 | 72 |
|  | **sPD-1** | **Correlation coefficient** | -0.077 | -0.105 |
|  |  | **Significance Level P** | 0.4468 | 0.3801 |
|  |  | **n** | 100 | 72 |
|  | **sPD-L1** | **Correlation coefficient** | -0.23 | -0.157 |
|  |  | **Significance Level P** | 0.0213 | 0.1887 |
|  |  | **n** | 100 | 72 |
|  | **sPD-L2** | **Correlation coefficient** | -0.085 | -0.017 |
|  |  | **Significance Level P** | 0.3992 | 0.8882 |
|  |  | **n** | 100 | 72 |
|  | **sTIM-3** | **Correlation coefficient** | 0.446 | 0.397 |
|  |  | **Significance Level P** | <0.0001 | 0.0006 |
|  |  | **n** | 100 | 72 |
|  | **sTLR-2** | **Correlation coefficient** | 0.204 | 0.066 |
|  |  | **Significance Level P** | 0.042 | 0.5829 |
|  |  | **n** | 100 | 72 |
| **CKs** | **CCL2** | **Correlation coefficient** | 0.439 | 0.23 |
|  |  | **Significance Level P** | <0.0001 | 0.0517 |
|  |  | **n** | 100 | 72 |
|  | **CCL3** | **Correlation coefficient** | 0.167 | 0.112 |
|  |  | **Significance Level P** | 0.1427 | 0.4238 |
|  |  | **n** | 78 | 53 |
|  | **CCL4** | **Correlation coefficient** | 0.419 | 0.335 |
|  |  | **Significance Level P** | 0.0002 | 0.0174 |
|  |  | **n** | 72 | 50 |
|  | **CXCL10** | **Correlation coefficient** | 0.369 | 0.109 |
|  |  | **Significance Level P** | 0.0002 | 0.3606 |
|  |  | **n** | 100 | 72 |
|  | **GM-CSF** | **Correlation coefficient** | 0.443 | 0.293 |
|  |  | **Significance Level P** | <0.0001 | 0.0232 |
|  |  | **n** | 81 | 60 |
|  | **IFN-α** | **Correlation coefficient** | -0.028 | -0.124 |
|  |  | **Significance Level P** | 0.8492 | 0.4838 |
|  |  | **n** | 48 | 34 |
|  | **IFN-γ** | **Correlation coefficient** | 0.012 | -0.103 |
|  |  | **Significance Level P** | 0.9157 | 0.4507 |
|  |  | **n** | 83 | 56 |
|  | **IL-10** | **Correlation coefficient** | 0.52 | 0.289 |
|  |  | **Significance Level P** | <0.0001 | 0.0147 |
|  |  | **n** | 99 | 71 |
|  | **IL-12p70** | **Correlation coefficient** | 0.346 | 0.222 |
|  |  | **Significance Level P** | 0.0005 | 0.0623 |
|  |  | **n** | 99 | 71 |
|  | **IL-13** | **Correlation coefficient** | 0.073 | 0.025 |
|  |  | **Significance Level P** | 0.529 | 0.8617 |
|  |  | **n** | 76 | 52 |
|  |  | **n** | 13 | 11 |
|  | **IL-1α** | **Correlation coefficient** | 0.202 | 0.148 |
|  |  | **Significance Level P** | 0.046 | 0.2206 |
|  |  | **n** | 98 | 70 |
|  | **IL-1β** | **Correlation coefficient** | 0.439 | 0.58 |
|  |  | **Significance Level P** | 0.0001 | <0.0001 |
|  |  | **n** | 74 | 54 |
|  | **IL-4** | **Correlation coefficient** | 0.451 | 0.483 |
|  |  | **Significance Level P** | 0.0009 | 0.0014 |
|  |  | **n** | 51 | 41 |
|  | **IL-6** | **Correlation coefficient** | 0.528 | 0.535 |
|  |  | **Significance Level P** | <0.0001 | <0.0001 |
|  |  | **n** | 87 | 62 |
|  | **IL-8** | **Correlation coefficient** | 0.437 | 0.27 |
|  |  | **Significance Level P** | <0.0001 | 0.0216 |
|  |  | **n** | 100 | 72 |
|  | **TNF-α** | **Correlation coefficient** | 0.529 | 0.298 |
|  |  | **Significance Level P** | <0.0001 | 0.023 |
|  |  | **n** | 84 | 58 |
